# Phytochemical Profile and Antidepressant Effect of *Ormosia henryi Prain* Leaf Ethanol Extract

**DOI:** 10.3390/ijms20143396

**Published:** 2019-07-10

**Authors:** Ying Lu, Shihao Zhu, Yingjie He, Changfu Peng, Zhi Wang, Qi Tang

**Affiliations:** 1 Hunan Key Laboratory of Traditional Chinese Veterinary Medicine, Hunan Agricultural University, Changsha 410128, China; 2 National Research Center of Engineering Technology for Utilization of Functional Ingredients from Botanicals, Hunan Agricultural University, Changsha 410128, China; 3 Hunan Linuo Biological Pharmaceutical Co. LTD, Guiyang 424400, China; 4 College of Pharmacy, Hunan University of Chinese Medicine, Changsha 410208, China

**Keywords:** HSCCC-prep-HPLC, UPLC-ESI-QTOF-MS/MS, flavone *C*-glycosides, CUMS mice, antidepressant effect, *Ormosia henryi Prain* leaf

## Abstract

The *Ormosia henryi Prain* leaf (OHPL) is a new bioactive resource with potential antidepressant activity, but few reports have confirmed its chemical composition or antidepressant effect. To investigate the phytochemical profile of OHPL ethanol extract (OHPLE), six flavone *C*-glycosides and two flavone *O*-glycosides were purified by high-speed counter-current chromatography combined with preparative high-performance liquid chromatography (HSCCC-prep-HPLC). The eight isolated compounds were identified by NMR and MS. Forty-six flavonoids, including flavones, flavone *C*-glycosides, flavone *O*-glycosides, isoflavones, isoflavone *O*-glycosides, prenylflavones and polymethoxyflavones were definitively or tentatively identified from OHPLE using ultra-performance liquid chromatography/ electrospray ionization quadrupole time-of-flight mass spectrometry (UPLC-ESI-QTOF-MS/MS) on the basis of fragment ions that are characteristic of these isolated compounds. The results of the antidepressant assay suggest that OHPLE significantly improved depression-related behaviors of chronic unpredictable mild stress (CUMS) mice. The observed changes in these mice after OHPLE treatment were an increased sucrose preference index, reduced feeding latency, prolonged tail suspension time, and upregulated expression of brain-derived neurotrophic factor (BDNF). The details of the phytochemicals and the antidepressant effect of OHPLE are reported here for the first time. This study indicates that the OHPL, enriched in flavone *C*-glycosides, is a new resource that might be potentially applied in the field of nutraceuticals (or functional additives) with depression-regulating functions.

## 1. Introduction

Depression-like diseases are complicated mental disorders characterized by a series of clinical symptoms, such as decreased interest, mood, and pleasure; and increased anxiety, sadness, stress, and anorexia [1,2,3]. Depression is treated with diverse clinical drugs, which are effective to a certain extent. Many antidepressant drug families, such as selective serotonin reuptake inhibitors, serotonin–norepinephrine reuptake inhibitors, tricyclic antidepressants, and monoamine oxidase inhibitors, work by enhancing the levels of neurotransmitters [4,5]. However, these synthetic antidepressants produce a lot of adverse effects, including serotonin syndrome, which is responsible for psychiatric disorders that induce behaviors such as irascibility, instability, distress, insomnia, and confusion [6,7]. Additional side effects include hypomania, hypertensive crisis, spontaneous abortion, and diminished libido [8,9,10]. Therefore, natural bioactive compounds with antidepressant activities and fewer side effects are needed as alternative depression treatments. Current studies have shown that many natural compounds or traditional herbal components can be used as clinical drugs or functional food sources for the treatment of depressive disorders. Natural plant sources produce secondary metabolites, such as flavonoids, coumarins, alkaloids, terpenoids, saponins, and polysaccharides, which have been proved to possess antidepressant activities [11]. Therefore, screening for low-toxicity, potent antidepressant compounds or components from natural plant sources is important for the development of novel nutraceuticals or health foods with depression-regulating functions. Such compounds include flavonoids, which have broad application value because of their antidepressant effects, low toxicities, and safety [8].

*Ormosia henryi Prain* (OHP), which belongs to the genus *Ormosia* (family Leguminosae), is a perennial green tree that is widely distributed in southern China. OPH roots, leaves, and stem bark have been applied as folk medicine to alleviate swallowing disorders, pain, and inflammation [12]. Clinical applications of traditional folk medicine have shown that OPH leaves possess a refreshing, invigorating, and antidepressant effect, suggesting that this plant has the potential for treating depression [13]. However, few studies have investigated the phytochemicals or pharmacological activities of OHP. Feng et al. conducted such a study, in which the constituents and the anti-inflammatory effect of OHP roots were assessed [12]. As a potential renewable resource, the phytochemical composition and antidepressant activity of the OHP leaf (OHPL) should be further studied.

Therefore, the main objectives of this study are to investigate the phytochemical profile and antidepressant effect of OHPL. To this end, OHPL was extracted by ethanol and eluted with macroporous resin (70% ethanol) to obtain OHPL ethanol extract (OHPLE). Eight flavonoids, including six flavone *C-*glycosides and two flavone *O-*glycosides, were separated and purified by combining high-speed counter-current chromatography and preparative high-performance liquid chromatography (HSCCC-prep-HPLC). The purified compounds were then characterized by NMR and MS. The characteristic fragment ions of the purified *C*-flavones and *O*-flavones were selected by ultra-performance liquid chromatography/electrospray ionization quadrupole time-of-flight mass spectrometry (UPLC-ESI-QTOF-MS/MS) to characterize or tentatively identify other homologous compounds in OHPLE, and the phytochemical constituents in OHPLE were systematically analyzed. Quantitative analysis was also carried out by using HPLC to measure the flavonoid compounds, and the results suggest that flavonoids are the dominant bioactive components of OHPLE. The antidepressant effect of OHPLE was evaluated by performing behavioral tests on chronic unpredictable mild stress (CUMS) mice. The sucrose preference test (SPT), ingestion latency test (ILT), and tail suspension test (TST) were conducted to identify changes in behavior after OHPLE treatment. Brain-derived neurotrophic factor (BDNF) expression in the hippocampus of test mice was also investigated. This study can be used as a reference for the development of OHPL as a new potential resource for antidepressant-related applications or a health functional food to decrease the risk of developing depression-like diseases.

## 2. Results

### 2.1. Isolation and Identification of Compounds **a**–**h**

**Figure 1:**
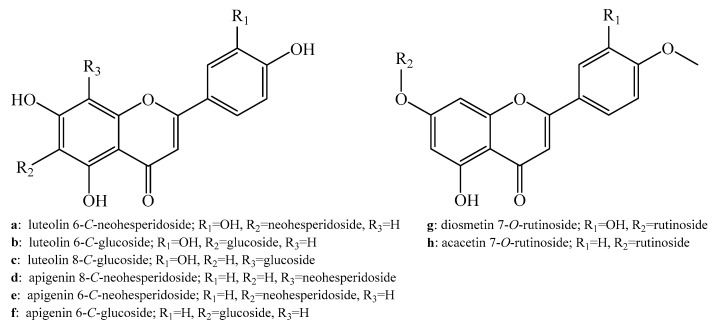
Structures of *compounds* ***a***–***h***.

The advantages of using HSCCC to isolate compounds from a complex mixture include high recovery, low consumption of solvents, and the non-denaturing and reversible adsorption of target compounds [14]. Consequently, HSCCC has been frequently employed as an efficient separation and preparative tool to isolate natural compounds from complicated mixtures of components. The key factors of HSCCC were successfully improved for the separation of the targets in this study, as detailed in Section 4.2. A combination of separation procedures using AB-8 macroporous resin and a prep-HPLC apparatus resulted in the efficient purification of eight compounds from OHPL (Figure 1). Six flavone *C-*glycosides and two flavone *O-*glycosides were identified by ^1^H- and ^13^C-NMR (Figure S1, Table S1) and MS. The ^1^H- and ^13^C-NMR results are specified in Table S1 and the details are listed below.

*Compound****a***: Yellowish powder, UV absorption at 270 and 352 nm, *m/z* 593.1515 [M−H]^−^. Identified as luteolin 6-*C*-neohesperidoside (isoorientin-2′′-*O*-rhamnoside) [15,16].

*Compound****b***: Yellowish powder, UV absorption at 270 and 350 nm, *m/z* 447.0931 [M−H]^−^. Identified as luteolin 6-*C*-glucoside (isoorientin) [17].

*Compound **c***: Yellow-green powder, UV absorption at 268 and 350 nm, *m/z* 447.0931 [M−H]^−^. Identified as luteolin 8-*C*-glucoside (orientin) [17,18].

*Compound****d***: Yellow-brown powder, UV absorption at 269 and 340 nm, deprotonated ion at *m/z* 577.1564 [M−H]^−^. Identified as apigenin 8-*C*-neohesperidoside (vitexin-2′′-*O*-rhamnoside) [19].

*Compound****e***: Yellow-brown powder, UV absorption at 270 and 340 nm, deprotonated ion at *m/z* 577.1562 [M−H]^−^. Identified as apigenin 6-*C*-neohesperidoside (isovitexin-2′′-*O*-rhamnoside) [20].

*Compound****f***: Light green powder, UV absorption at 270 and 338 nm, deprotonated ion at *m/z* 431.0985 [M−H]^−^. Identified as apigenin 6-*C*-glucoside (isovitexin) [21,22].

*Compound****g***: White powder, UV absorption at 266 and 348 nm, *m/z* 607.1783 [M−H]^−^. Identified as diosmetin 7-*O*-rutinoside (diosmin) [23].

*Compound****h***: White powder, UV absorption at 269 and 332 nm, *m/z* 591.1880 [M−H]^−^. Identified as acacetin 7-*O*-rutinoside (linarin) [24,25].

It is noteworthy that flavone *C-*diglycosides, such as luteolin 6-*C*-neohesperidoside, exhibited double NMR signals because the disaccharide substituent differs in spatial conformation, resulting in different structures [20]. Results suggest the presence of two conformers of flavone *C-*diglycosides.

### 2.2. Phytochemical Investigation of OHPLE using UPLC-ESI-QTOF-MS/MS 

**Figure 2:**
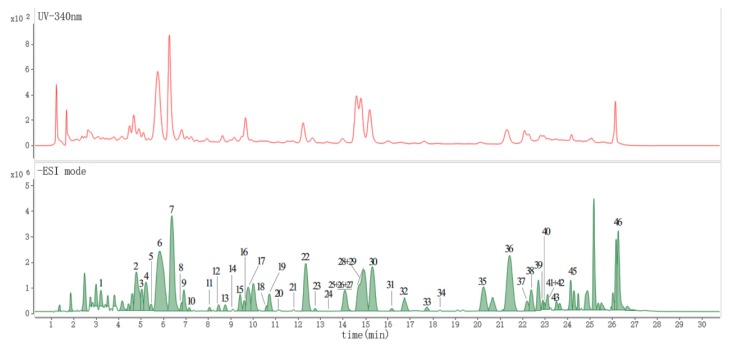
UPLC‒UV chromatogram (340 nm) and UPLC-ESI-QTOF-MS/MS base peak chromatogram (BPC) in negative ESI mode of OHPLE.

For the characterization of other homologous compounds, the eight compounds isolated from OHPLE were selected as reference standards to analyze their MS fragmentation patterns. The UPLC‒UV chromatogram (340 nm) and UPLC-ESI-QTOF-MS/MS base peak ion chromatogram of OHPLE are shown in Figure 2. By careful deduction, 46 flavonoids (Table 1) were definitively or preliminarily characterized by comparing their precise molecular weights, retention times, ultraviolet spectra, splitting patterns, and fragment ions with those of the reference compounds or values in the literature.

**Figure 3:**
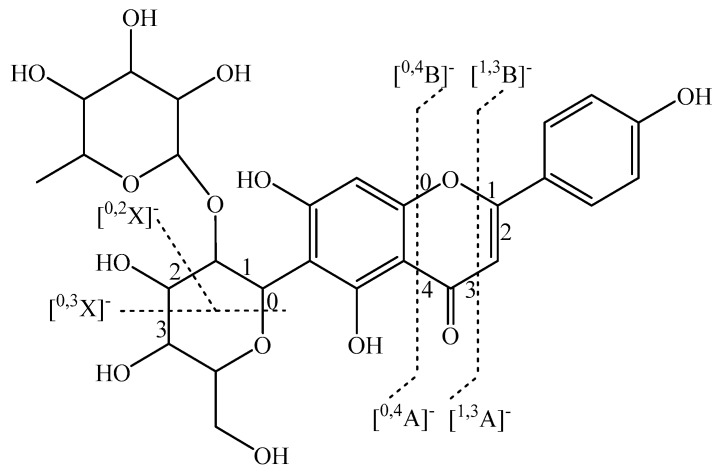
Nomenclature of fragments (apigenin 6-*C*-neohesperidoside is shown as an example).

In particular, we elaborate on the main components of flavone *C*-glycosides. In previous studies, flavone *C*-glycosides have usually been found at positions C-6 and/or C-8 [26], and this was also observed for OHPLE. Flavone *C*-glycosides were found to be the main components in OHPLE. The general nomenclature of the fragments is shown in Figure 3. 

**Figure 4:**
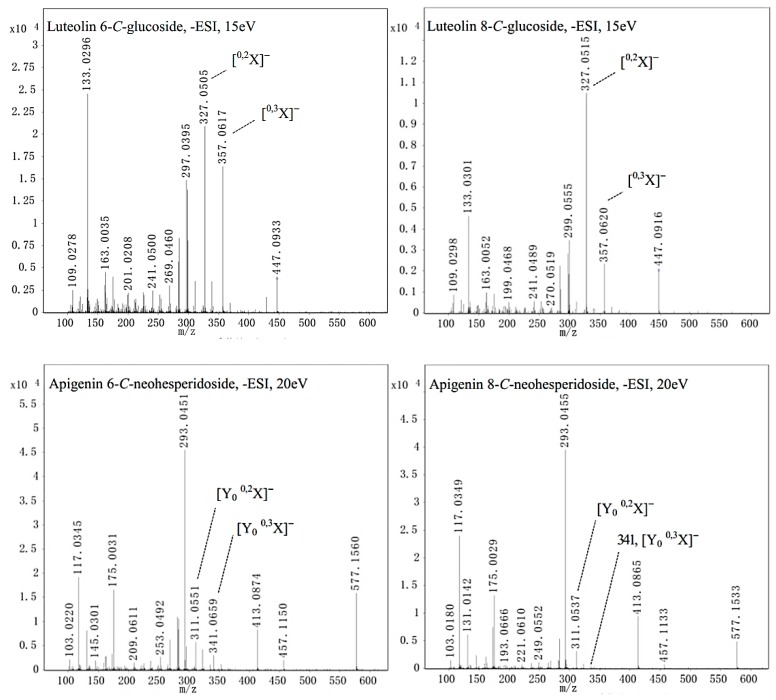
MS fragments of two pairs of *C*-flavone isomers.

The flavone *C*-monoglycoside isomers luteolin 6-*C*-glucoside (***b***) and luteolin 8-*C*-glucoside (***c***) and the flavone *C*-diglycoside isomers apigenin 6-*C*-neohesperidoside (***e***) and apigenin 8-*C*-neohesperidoside (***d***) are shown in Figure 4 as examples of flavone *C*-glycosides to illustrate the typical fragmentation patterns found by UPLC-ESI-QTOF-MS/MS. 

Briefly, flavone *C*-diglycoside was first cleaved into an ion of flavone *C*-monoglycoside by the loss of a rhamnoside (Rha) [M−H−146]^−^ at low collision energy (5 eV). From the loss of Rha, [^0,3^X]^−^ ([M−H−146−90]^−^) and [^0,2^X]^−^ ([M−H−146−120]^−^) fractures were the dominant fragmentation pathways at increased energy (20 eV). The relative abundance ratio of [^0,3^X]^−^ and [^0,2^X]^−^ was used as a critical index to discriminate between *C-*6 and *C*-8 glycosides. The compound was identified as a *C*-6 glycoside when [^0,3^X]^−^/[^0,2^X]^−^ was greater than 1/2. If [^0,3^X]^−^/[^0,2^X]^−^ was less than or close to 1/5, the compound was designated as a *C*-8 glycoside. The main fragmentation pathways of flavone *C*-monoglycosides were [^0,3^X]^−^ ([M−H−90]^−^) and [^0,2^X]^−^ ([M−H−120]^−^) cleavages, which followed the same pattern of flavone *C*-diglycoside cleavages. The other flavone *C*-glycosides isolated from OHPL exhibited results consistent with this pattern. Dehydration ([M−H−146−18]^−^) was also carried out, and multiple product ions were formed at higher collision energies (15 and 20 eV); these could be critical ions for determining the structure of aglycones. In short, these compounds were definitively or tentatively identified from OHPLE according to the particular fragmentation patterns. The other compounds, including flavones, flavone *O*-glycosides, isoflavones, and isoflavone *O*-glycosides were also characterized. The detailed description can be found in the supplemental data.

### 2.3. Quantitative Analysis of OHPL and OHPLE 

Quantitative analysis was performed on the basis of the calibration curves of eight flavone standards by using HPLC detection, as depicted in Table 2. Similar to OHPL, the results suggest that OHPLE was enriched with flavonoids (151.54 ± 1.19 mg/g). *Compound **d*** apigenin 8-*C*-neohesperidoside (33.63 ± 0.49 mg/g) and *compound* ***e*** apigenin 6-*C*-neohesperidoside (47.43 ± 0.18 mg/g) were the dominant *C-*flavones in OHPLE.

### 2.4. OHPLE Alleviated Depression symptoms in CUMS Mice

**Figure 5:**
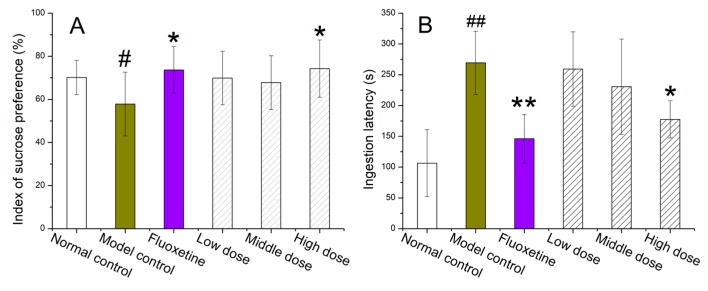

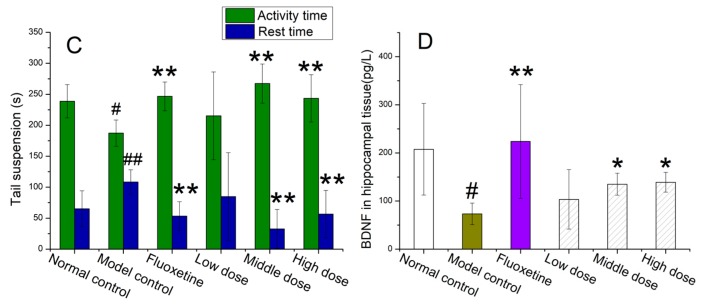
The effects of a series of OHPL extract doses on the behaviors of CUMS mice after treatment. (**A**) Sucrose preference test, (**B**) ingestion latency test, (**C**) tail suspension test and (**D**) brain-derived neurotrophic factor expression. The values are expressed as the mean ± SEM. For statistical significance, # *p* < 0.05, ## *p* < 0.01 compared with the normal control group; * *p* < 0.05, ** *p* < 0.01 compared with the model control group.

As shown in Figure 5A, the SPT index of mice in the model control group was markedly decreased (*p* < 0.05) compared with the normal control group, suggesting that the CUMS mouse model was successfully established. Compared with the model control group, the SPT index of mice in different OHPLE dose groups (low, medium, and high) and the fluoxetine group increased by 20.9% (low dose), 17.3% (medium dose), 28.5% (high dose), and 27.4%, respectively. The increase in the SPT index in the high-dose group was significant (*p* < 0.05). 

Figure 5B shows the ILT results, which reveal that the ingestion latency time of mice in the model control group was significantly prolonged compared with that of the mice in the normal control group (*p* < 0.01). The ingestion latency time of mice in the different OHPLE dose groups and the fluoxetine group decreased by 3.7% (low dose), 15.0% (medium dose), 34.2% (high dose), and 45.7%, respectively. The decreases in ILT values in the high-dose OHPLE group and fluoxetine group were significant (*p* < 0.05, *p* < 0.01). 

Figure 5C shows the TST results, which show that the activity time of mice in the model control group was significantly reduced (*p* < 0.05) compared with the normal control group, and the rest time was significantly increased (*p* < 0.01). The activity time of mice in different OHPLE dose groups and the fluoxetine group was prolonged by 15.0% (low dose), 42.8% (medium dose), 29.9% (high dose), and 31.6%, respectively, and the increases in activity time in three groups (medium dose, high dose, and fluoxetine groups) were significant (*p* < 0.01). At the same time, the rest time of the mice decreased by 21.3% (low dose), 70.3% (medium dose), 47.2% (high dose), and 50.9%, respectively. The decreases in rest time in three groups (medium-dose, high-dose, and fluoxetine groups) were significant (*p* < 0.01).

Figure 5D shows that the BDNF content in the hippocampal tissue of mice in the model control group was significantly reduced (*p* < 0.01) compared with that in the normal control group. The BDNF content in different OHPLE dose groups and the fluoxetine group increased by 41.3% (low dose), 84.1% (medium dose), 89.5% (high dose), and 205.2%, respectively, and the increases in BDNF levels in three groups (medium-dose, high-dose, and fluoxetine groups) were significant (*p* < 0.05, *p* < 0.01). 

The above results show that the high dose of OHPLE significantly increased the index of sucrose preference and reduced the ingestion latency, medium and high doses of OHPLE significantly prolonging the activity time and reducing the rest time. The BDNF expression in the medium-dose and high-dose groups was significantly increased.

## 3. Discussion

Few phytochemical studies of *Ormosia* genus have been reported. Among these reports, oils [27], quinolizidine alkaloids [28,29], and phenols [30] were proved to exist in this genus. In the current study, the phytochemical investigation and quantitative analysis suggest that flavonoids are the dominant antidepressant components in OHPLE, and it has significant antidepressant effects on CUMS mice.

Antidepressant effects of flavonoids have been performed by many animal model experiments [8,31], and the mechanisms of flavonoids and related analogues are on neurotransmitters, including 5-HT, NA, and DA [32]. *C*-flavones with monoglycoside or diglycoside possess antidepressant effects [3]. The flavonoid fraction of *Cecropia pachystachya* was reported to mainly contain luteolin 6-*C*-glucoside (isoorientin), luteolin 8-*C*-glucoside (orientin), and apigenin 6-*C*-glucoside (isovitexin) which could reduce the immobility time in the FST (forced swimming test) at doses of 50 and 100 mg/kg. These results suggested that the flavone *C*-glycoside-enriched fraction exerts antidepressant-like effects [33]. Vitexin (apigenin-8-*C*-glucoside) significantly increased the mobility time in the FST and TST of mice and exhibited antidepressant activity. The mechanisms of vitexin’s antidepressant effects were revealed to be an increase in the catecholamine content in the synaptic cleft, and it interacted with serotonergic 5-HT_1A_; noradrenergic α_2_; and dopaminergic D_1_, D_2_, and D_3_ receptors [34]. Flavones with an approximated structure, e.g., hydroxy groups, such as luteolin and apigenin, were shown to have good antidepressant capacity through their modulation of monoamine oxidase activity [35]. Oral administration of luteolin (at a dose of 50 mg/kg) resulted in antidepressant effects in some behavioral tests (in the FST and TST) [36]. Luteolin raised the levels of monoamine neurotransmitters in the synaptic cleft by directly and indirectly inhibiting serotonin reuptake [37]. Apigenin at doses of 12.5 and 25 mg/kg displayed antidepressant-like effects by significantly increasing the mobility of mice in the FST. Furthermore, apigenin administration increased mobility in the FST, induced a decrease in DA turnover in the amygdala, and increased DA turnover in the hypothalamus at a dose of 25 mg/kg [38]. Apigenin also attenuated CMS-induced alterations in serotonin (5-HT) [39]. Moreover, the isoflavones genistein and daidzein resulted in an increased activity time in the FST, indicating an effective antidepressant effect in mice [40]. These data support the results of the antidepressant effect of OHPLE in the current study directly or indirectly. 

## 4. Materials and Methods

### 4.1. Plant Material and Reagents

*Ormosia henryi Prain* leaf (OHPL) samples (No. 20180809) were obtained from Hunan Linuo Biological Pharmaceutical Co., Ltd. (Chenzhou, China) and identified by Zhi Wang (College of Pharmacy, Hunan University of Traditional Chinese Medicine). Organic reagents, including *n*-butanol, methyl tert-butyl ether (MTBE) ethanol, acetic acid, and hydrochloric acid were used for sample preparation and isolation. Analytical-grade acetonitrile, methanol, and formic acid (Sinopharm Chemical Reagent Co., Ltd., Shanghai, China) were used for HPLC and UPLC-ESI-QTOF-MS/MS analysis. Ultra-pure water was obtained from a hyperpure water purifier (Barnstead Easy Pure II, Merck chemical technology, Co. Ltd., Shanghai, China).

### 4.2. Extraction and Isolation of Dominant Compounds

For extract preparation, 2.0 kg of dried OHPL was ultrasonically extracted with 30.0 L of 70% ethanol for 30 min in triplicate. The filtered solutions were gathered and concentrated by a rotary evaporator (R1001, Zituo instrument equipment Co., Ltd., Zhengzhou, China) under reduced pressure at 55 °C until there was no ethanol odor. The concentrate was obtained and then extracted by petroleum ether until the ether layer was colorless. Then, the pH of the OHPL concentrate was adjusted to 3 by adding hydrochloric acid. About 6.0 L of OHPL concentrate was passed through a column containing 1000 mL of AB-8 macroporous resin at 2–3 BV/h and left to stand for 30 min. This resin was washed by ultrapure water (about 10 BV) and then eluted with 70% ethanol at a flow speed of 2–3 BV/h. The 70% ethanol eluent was collected and concentrated by a rotary evaporator under reduced pressure at 55 °C until there was no smell of ethanol. It was dried under vacuum, and a freeze-drying apparatus (MODULYOD-230, Thermo Fisher Scientific, Waltham, MA, USA) was used to obtain OHPLE. The weight of OHPLE was 50.15 g, and it was prepared for subsequent HSCCC separation and antidepressant experiments.

HSCCC (Model TBE 300A, Tauto Biotechnique Co., Ltd., Shanghai, China) [41] was used for the purification of OHPLE. Optimal conditions were determined on the basis of values in the literature [42,43] and the properties of these compounds. The optimal conditions were determined to be a rotation rate of 850 rpm, mobile phase flow rate of 2.0 mL/min, multilayer-coiled column temperature of 20 °C, and detection wavelength of 340 nm. The solvent system composed of *n*-butanol–MTBE–ethanol–0.1% acetic acid (3:1:1:6, *v*/*v*) which produced good *K*-values for the target compounds and was selected as the separation system in the HSCCC experiment.

**Figure 6:**
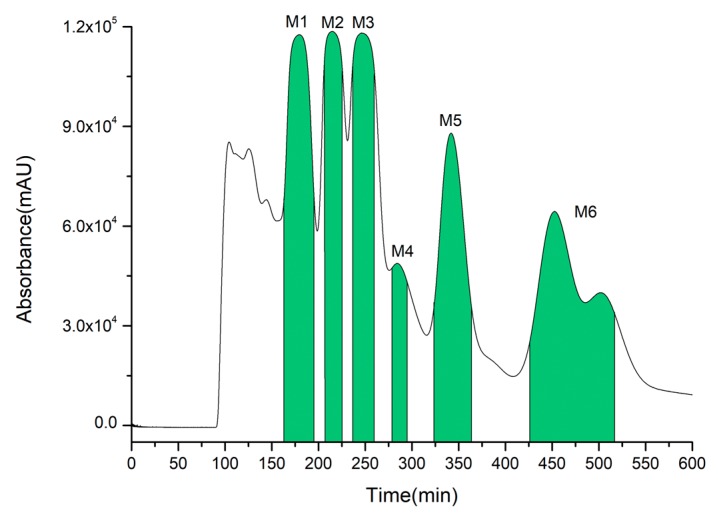
HSCCC chromatogram of OHPLE. Solvent system: *n*-butanol–MTBE–ethanol–0.1% acetic acid (3:1:1:6, *v*/*v*). The detection wavelength was 340 nm. Sample loading was 250 mg in 20 mL of the lower phase. The rotation speed was 850 rpm. The temperature of the separation columns was maintained at 20 °C, and the flow rate of the mobile phase was 2.0 mL/min.

OHPLE (250 mg) was dissolved in the lower phase (20 mL) for HSCCC separation, and the same procedure was repeated 20 times (total OHPLE mass of 5.0 g). As illustrated in Figure 6, six fractions from OHPLE (M1, 162–193 min; M2, 204–226 min; M3, 238–261 min; M4, 278–293 min; M5, 323–363 min; M6, 426–521 min) were collected and dried individually. Two compounds, M2 (*compound **4***, 254.1 mg) and M3 (*compound* ***5***, 234.4 mg), were obtained with purities of over 98% according to HPLC analysis. The other four fractions were not pure and required further purification by prep-HPLC with an LC-8A system [44] and HPCL C18 column (250 × 10 mm, 5 µm). These compounds were purified by prep-HPLC using a solvent system of acetonitrile and 0.1% acetic acid in water. The flow rate was 6.0 mL/min, and the process was monitored at a 340 nm wavelength. The compounds were separated by prep-HPLC with purities of over 98%. M1 contained *compound **1*** (12.3 mg), M4 contained *compound **7*** (6.9 mg), M5 contained *compound **2*** (24.5 mg) and ***3*** (31.6 mg), and M6 contained *compounds **6*** (11.2 mg) and ***8*** (14.1 mg). 

### 4.3. UPLC-ESI-QTOF-MS/MS for Phytochemical Analysis

An Agilent 1290 HPLC system (Agilent Technologies, Palo Alto, CA, USA) with an Agilent 6530 QTOF-MS mass spectrometer was employed for the phytochemical analysis of OHPLE [45]. Matrix separation was performed using an XAqua C18 column (2.1 × 150 mm, 5 µm, Agilent Technologies, Acchrom Technologies Co., Ltd., Beijing, China) at a constant temperature (30 °C) and detected at 340 nm. Deionized water (0.1% formic acid, A) and acetonitrile (B) were selected as the solvent system with an optimized gradient elution procedure (0~20 min, 10~90% B) at a flow rate of 0.3 mL/min. The injection volume was 2 µL (1.0 mg/mL). The parameters of the MS system were a capillary voltage of 3.5 kV for negative mode, a nebulizer pressure of 50 psi, and a nozzle voltage of 1.0 kV. The drying gas flow rate was 6 L/min, the sheath gas temperature was 350 °C with a flow rate of 11 L/min, the skimmer voltage was 65 V, OCT1 RF Vpp was 750 V, and the fragmentor voltage was 135 V. The mass data were acquired in negative and positive mode and ranged from *m/z* 100 to 1000 Da. The typical MS^2^ fragment ions of the isolated compounds were obtained by multiple collision energies, which varied from 5 to 40 eV. Mass Hunter Qualitative Analysis B.08.00 was employed for the data analysis.

### 4.4. Quantitation of Isolated Compounds

The quantitation of the eight compounds isolated from OHPLE was performed by a LA-20AT HPLC system (Shimadzu Corporation, Kyoto, Japan) coupled with a GL Sciences WondaSil C18 column (4.6 × 250 mm, 5 µm) with a detection wavelength of 340 nm. The column temperature was 30 °C and the injection volume was 20 μL, and 0.2% phosphoric acid (A) and acetonitrile (B) were selected as the solvent system at a flow rate of 1.0 mL/min and the following gradient elution procedure: B (15–23%) for 0–8 min, B (23–30%) for 8–20 min, B (30–60%) for 20–25 min, and B (60–15%) for 25–26 min. Calibration curves were obtained by detecting a series of concentrations of the target compounds. Then, the content of each compound (mg/g) was calculated.

### 4.5. Model of CUMS Mice

Male ICR mice (weight range of 18.0–22.0 g) were purchased from Hunan Slake Jing-da Experimental Animals Co., Ltd. (Certificate number 43004700048590). In the barrier environment of the Hunan Drug Safety Evaluation and Research Center, the mice were fed at constant temperature (22 ± 2 °C) with a 12-h light/dark cycle (lights on at 8:00 a.m., lights off at 8:00 p.m.). The laboratory animal use license number is SYXK 2015-06. All experiments and procedures were carried out according to the Regulations of Experimental Animal Administration issued by the State Committee of Science and Technology of China.

In addition to the normal control group of 10 mice, the CUMS model was induced in 60 mice by the following methods: (1) food deprivation for 12 h, (2) water deprivation for 12 h, (3) forced swimming for 10 min, (4) strobe flash for 12 h, (5) noise for 30 min, (6) physical restraint for 12 h while deprived of food and water (placed in a 50 mL centrifugal tube with a diameter of 3 cm, length of about 10 cm, and 6–7 vents in the tube wall with a diameter of 0.5 mm), (7) tilt the cage for 12 h, (8) wet the cage for 12 h, and (9) reversed day and night. These forced experiments were scheduled to take place within a week and repeated for 4 weeks. The normal control mice were fed in cages without disturbance except for cage cleaning.

### 4.6. Drug Administration

The normal control group was treated with distilled water. There were five CUMS mice groups. One group received the positive control drug fluoxetine (5.2 mg/kg), a widely used antidepressant, which was purchased from Ritual Suzhou Pharmaceutical Co., Ltd. (Suzhou, China). The clinical dose of fluoxetine was 20 mg, which was converted into the appropriate dose for mice according to the clinical dose of animals and humans: 20 mg × 0.0026/0.02 kg = 2.6 mg/kg. The clinical equivalent dose was twice that calculated for mice, namely, 5.2 mg/kg. The other three groups were administered with different doses of OHPLE: a low-dose group (50.0 mg/kg/day), medium-dose group (100.0 mg/kg/day), and high-dose group (150.0 mg/kg/day). For the model control group, the mice were supplied with the equivalent volume of distilled water instead of the treatments. Each group was treated the same way for 35 consecutive days to complete their corresponding treatments. The route of administration was gavage. After the last administration, the mice were assessed using the sucrose preference test (SPT), ingestion latency test (ILT), and tail suspension test (TST), and BDNF expression was measured. Results of the acute toxicity test did not reveal any toxicity up to an OHPLE dose of 10.0 g/kg/day.

### 4.7. Behavior Tests

The SPT was divided into a training period and test period. The first 24 h was used as the training period to allow the animals free access to two bottles of a 1% sucrose solution. In the subsequent 24 h, one of the bottles was replaced with pure water. Mice were disallowed water but not food for 8 h before the test. The SPT was performed at 9:00 a.m. Mice were housed individually and given free access to a bottle of 100 mL of a 1% sucrose solution and a bottle of 100 mL of pure water. After 15 h, the consumption of the sucrose solution and the pure water was calculated (during the test, the positions of the two bottles were swapped to eliminate the influence of location preference). The sucrose preference was calculated by the following formula: sucrose preference = sucrose consumption/(pure water consumption + sucrose consumption) × 100%.

The ILT was performed after the SPT. In brief, on the first day, mice were placed in a square box for 10 min to allow adaptation. Afterward, the mice were deprived of food but not water for 24 h. During the test, a food pellet was placed in the center of the open box, and the mice were put back to access the food pellet (mice were placed in the same position and direction each time). The time between when the mouse was put into the cage and the first time it fed (each time lasted for 5 min) was observed and recorded.

The TST was performed after the ILT. In this test, mice were suspended by their tail on a tail suspension device. The head was about 5 cm from the table so that there was no place for the mice to climb onto or grasp. The activity time and rest time of the mice were recorded within the remaining 4 min. Mice were prevented from seeing one another to prevent mutual interference.

Finally, the tested mice were sacrificed by cervical dislocation. Hippocampal tissues were quickly taken, and the mean BDNF expression of each group was determined by an immunofluorescence technique. In short, hippocampal tissue was homogenized and centrifuged at 1000 rpm for 10 min. Then, the supernatant was taken, and the BDNF content was detected at 540 nm (Spectra Max i3x multifunctional spectrum, Meigu Molecular Instruments Co., LTD, Shanghai, China) using an Elisa kit (No. 18072, Wuhan Genmei Biotechnology Co. LTD, Wuhan, China).

### 4.8. Statistical Analysis

SPSS (version 16.0) was employed for statistical analysis, and the measurement data were expressed by the mean ± SEM. The data of all groups were analyzed by a one-way analysis of variance (ANOVA) followed by Dunnett’s test in order to detect inter-group differences. *p* < 0.05 was considered as statistically significant.

## 5. Conclusions

In this study, the detailed chemical profile and the preliminary antidepressant effect of OHPLE are described for the first time. The results suggest the potential application of OHPLE (rich in flavone *C*-glycosides) in the field of nutraceuticals and as functional food additives with depression-regulating functions. 

## Figures and Tables

**Table 1:** Identification of 46 flavonoids from OHPLE by UPLC-ESI-QTOF-MS/MS.

Peak	RT (min)	λ_max_ (nm)	[M−H]^−^ (*m/z*)	Formula	ppm	MS^2^ fragment ions (*m/z*)	Identification	Ref.
**Flavone *C*-glycosides**								
2	4.7	270, 352	593.1523	C27H30O15	-1.97	447 [M−H−Rha]^−^, 357 [^0,3^X]^−^, 327 [^0,2^X]^−^, 285, 163, 133	luteolin 6-*C*-neohesperidoside ^※^	[15,16]
3	5.1	270, 350	447.0936	C21H20O11	-1.31	357 [^0,3^X]^−^, 327 [^0,2^X]^−^, 285, 175, 163, 133	luteolin 6-*C*-glucoside ^※^	[17]
4	5.2	268, 350	447.0939	C21H20O11	-1.90	357 [^0,3^X]^−^, 327 [^0,2^X]^−^, 285, 175, 163, 133	luteolin 8-*C*-glucoside ^※^	[17,18]
5	5.4	270, 352	593.1527	C27H30O15	-2.03	447 [M−H−Rha]^−^, 357 [^0,3^X]^−^, 327 [^0,2^X]^−^, 285; 163; 133	luteolin 8-*C*-neohesperidoside ^‡^	
6	5.8	269, 340	577.1573	C27H30O14	-1.56	431 [M−H−Rha]^−^, 341 [^0,3^X]^−^, 311 [^0,2^X]^−^, 283, 269, 175, 131, 117	apigenin 8-*C*-neohesperidoside ^※^	[19]
7	6.4	270, 340	577.1574	C27H30O14	-1.74	431 [M−H−Rha]^−^, 341 [^0,3^X]^−^, 311 [^0,2^X]^−^, 283, 269, 175, 131, 117	apigenin 6-*C*-neohesperidoside ^※^	[20]
8	6.7	271, 340	593.1526	C27H30O15	-2.18	431 [M−H−Glc]^−^, 341 [^0,3^X]^−^, 311 [^0,2^X]^−^, 283, 269, 175, 131, 117	apigenin 8-*C*-diglucoside ^‡^	
9	6.8	270, 338	431.0981	C21H20O10	0.34	341 [^0,3^X]^−^, 311 [^0,2^X]^−^, 283, 269, 117	apigenin 6-*C*-glucoside ^※^	[21,22]
15	9.4	/	433.1141	C21H22O10	0.13	343 [^0,3^X]^−^, 271	naringenin *C*-glucoside ^‡^	
**Flavones**								
18	10.4	287	271.0613	C15H12O5	-1.01	/	naringenin isomer ^‡^	
19	10.7	286	287.0565	C15H12O6	-1.7	151 [^1,3^A]^−^, 135 [^1,3^B]^−^	aromadendrin ^‡^	[12]
26	14.1	266, 323	285.0408	C15H10O6	-1.49	151 [^1,3^A]^−^, 133 [^1,3^B]^−^	kaempferol ^‡^	[12]
27	14.4	288, 324	287.0563	C15H12O6	-0.72	151 [^1,3^A]^−^, 135 [^1,3^B]^−^	eriodictyol ^‡^	
31	16.1	269, 328	283.0613	C16H12O5	0.17	151 [^1,3^A]^−^, 131 [^1,3^B]^−^	acacetin isomer ^‡^	
32	16.7	266, 335	285.0409	C15H10O6	-1.48	151 [^1,3^A]^−^, 133 [^1,3^B]^−^	luteolin	
35	20.2	288	271.0609	C15H12O5	0.93	151 [^1,3^A]^−^, 119 [^1,3^B]^−^	naringenin ^‡^	[12]
37	22.1	270, 330	283.0609	C16H12O5	-2.21	151 [^1,3^A]^−^, 131 [^1,3^B]^−^	acacetin ^‡^	
38	22.3	263	269.0459	C15H10O5	-1.43	151 [^1,3^A]^−^, 117 [^1,3^B]^−^	apigenin ^‡^	[12]
43	23.5	266	299.0566	C16H12O6	-1.24	151 [^1,3^A]^−^, 147 [^1,3^B]^−^	diosmetin ^‡^	
44	23.7	/	299.0568	C16H12O6	-1.93	/	diosmetin isomer ^‡^	
**Flavone *O*-glycosides**								
1	3.2	287	595.1675	C27H32O15	-1.42	287 [M−H−rutinoside]^−^, 241, 213, 151, 117	aromadendrin 3*-O*-rutinoside ^‡^	
10	7.1	267, 338	577.1571	C27H30O14	-2.56	431[M−H−Rha]^−^, 269 [M−H− rutinoside]^−^, 151, 117	apigenin *O*- rutinoside ^‡^	
12	8.4	/	579.2093	C28H36O13	-1.65	417 [M−H−Glc]^−^, 271 [M−H−Glc−Rha]^−^, 151, 119	naringenin *O*-rha-glucoside ^‡^	
16	9.5	260	^#^433.1124	C21H20O10	1.09	271[M+H−Glc]^+^, 153, 119	apigenin 7-*O*-glucoside ^‡^	
17	9.7	266, 348	607.1679	C28H32O15	-1.66	299 [M−H−rutinoside]^−^, 151, 147	diosmetin 7-*O*-rutinoside ^※^	[23]
28	14.6	268, 325	577.1575	C27H30O14	-1.96	445 [M−H−pentoside]^−^, 283 [M−H−pentoside−Glc]^−^, 151, 131	acacetin *O*-glc-pentoside ^‡^	
29	14.8	269, 332	591.1713	C28H32O14	1.13	283 [M−H−rutinoside]^−^, 151, 131	acacetin 7-*O*-rutinoside ^※^	[24,25]
30	15.2	268, 325	577.1577	C27H30O14	-2.19	445 [M−H−pentoside]^−^, 283[M−H−pentoside−Glc]^−^, 151, 131	acacetin *O*-glc-pentoside ^‡^	
**Isoflavones**								
22	12.2	259	283.0611	C16H12O5	0.33	255 [M−H−CO]^−^, 227 [M−H−2CO]^−^, 151 [^1,3^A]^−^, 131 [^1,3^B]^−^	biochanin A ^‡^	[12]
25	13.9	258	253.0510	C15H10O4	-1.63	225 [M−H−CO]^−^, 197 [M−H−2CO]^−^, 135 [^1,3^A]^−^, 117 [^1,3^B]^−^	daidzein ^‡^	[12]
36	21.4	260	269.0457	C15H10O5	-0.62	241 [M−H−CO]^−^, 213 [M−H−2CO]^−^, 151 [^1,3^A]^−^, 117 [^1,3^B]^−^	genistein ^‡^	[12]
45	24.2	259	267.0660	C16H12O4	0.70	239 [M−H−CO]^−^, 211 [M−H−2CO]^−^, 149 [^1,3^A]^−^, 117 [^1,3^B]^−^	isoformononetin ^‡^	[12]
46	26.2	261	283.0613	C16H12O5	-0.52	255 [M−H−CO]^−^, 227 [M−H−2CO]^−^, 165 [^1,3^A]^−^, 117 [^1,3^B]^−^	isoprunetin ^‡^	[12]
**Isoflavone *O*- glycosides**								
11	8.0	/	431.0981	C21H20O10	-2.19	269 [M−H−Glc]^−^, 213, 151	genistein7-*O*-glucoside ^‡^	
13	8.7	264, 327	577.1571	C27H30O14	-2.56	431[M−H−Rha]^−^, 269 [M−H− rutinoside]^−^, 241, 213, 151, 117	genistein7-*O*-rutinoside ^‡^	
14	9.0	/	^#^593.1867	C28H32O14	0.02	285 [M+H− rutinoside]^+^, 229, 153	biochanin A -*O*-rutinoside ^‡^	
21	11.7	265, 325	577.1572	C27H30O14	-1.71	445 [M−H−pentoside]^−^, 283[M−H−pentoside−Glc]^−^, 255, 227, 165, 117	isoprunetin 7-*O*-glc-pentoside ^‡^	
33	17.7	261, 325	^#^447.1290	C22H22O10	-0.62	285 [M+H−Glc]^+^, 257, 229, 167, 119	isoprunetin 7-*O*-glucoside ^‡^	[12]
**Prenylflavones and Polymethoxyflavones**								
23	12.7	/	339.1247	C20H20O5	-2.20	/	prenylnaringenin ^‡^	
34	18.3	/	355.1196	C20H20O6	0.37	/	prenylaromadendrin ^‡^	
20	11.1	/	^#^375.1446	C20H22O7	-1.81	360 [M+H−CH_3_]^+^	pentamethoxyflavanone ^‡^	
24	13.4	/	^#^315.0854	C17H14O6	2.50	300 [M+H−CH_3_]^+^, 285 [M+H−2CH_3_]^+^	dihydroxyl-dimethoxyflavone ^‡^	
39	22.8	/	297.0778	C17H14O5	-1.79	282 [M−H−CH_3_]^−^, 267 [M−H−2CH_3_]^−^	dimethoxyl-hydroxyflavone ^‡^	
40	22.9	/	297.0770	C17H14O5	-0.59	282 [M−H−CH_3_]^−^, 267 [M−H−2CH_3_] ^−^	dimethoxyl-hydroxyflavone ^‡^	
41	23.1	/	373.0926	C19H18O8	0.99	343 [M−H−2CH_3_]^−^	tetramethoxyl-dihydroxyflavone ^‡^	
42	23.2	/	373.0925	C19H18O8	0.97	343 [M−H−2CH_3_]^−^	tetramethoxyl-dihydroxyflavone ^‡^	

^※^ These compounds were isolated by HSCCC-prep-HPLC; ^‡^ these compounds were tentatively identified by QTOF-MS; ^#^ these values were obtained in positive mode.

**Table 2:** Quantitative analysis of the dominant compounds in OHPL and OHPLE (mg/g).

	*a*	*b*	*c*	*d*	*e*	*f*	*g*	*h*	Total
OHPL	0.81 ± 0.11	0.51 ± 0.14	0.36 ± 0.10	1.26 ± 0.07	2.23 ± 0.28	0.57 ± 0.11	0.94 ± 0.23	0.86 ± 0.75	7.53 ± 0.45
OHPLE	12.87 ± 0.18	7.77 ± 0.49	6.25 ± 0.40	33.63 ± 0.49	47.43 ± 0.18	5.94 ± 0.04	13.25 ± 0.38	24.39 ± 0.22	151.54 ± 1.19

## Supplementary Materials

Supplementary materials can be found at https://www.mdpi.com/1422-0067/20/14/3396/s1.

## Abbreviations

OHPL	Ormosia henryi Prain leaves
OHPLE	Ormosia henryi Prain leaf ethanol extract
CUMS	chronic unpredictable mild stressed
SPT	sucrose preference test
ILT	ingestion latency test
TST	tail suspension test
BDNF	brain-derived neurotrophic factor
HSCCC	high-speed counter-current chromatography
prep-HPLC	preparative high-performance liquid chromatography
UPLC-ESI-QTOF-MS/MS	ultra-performance liquid chromatography/electrospray ionization quadrupole time-of-flight mass spectrometry
