# Supplementary material for: Phytochemical Profile and Antidepressant Effect of Ormosia henryi Prain Leaf Ethanol Extract
Source: Int J Mol Sci. 2019 Jul 10;20(14):3396. doi: 10.3390/ijms20143396 (PMC6678957; doi:10.3390/ijms20143396)
Supplement: Supplementary file 1 [file ijms-20-03396-s001.pdf]

Supplemental Material

Figure S1. The detailed ^{1}H- and ^{13}C-NMR data of isolated compounds

Compound ***a*** Luteolin 6-*C*-neohesperidoside (isoorientin-2″-*O*-rhamnoside)

^{1}H-NMR

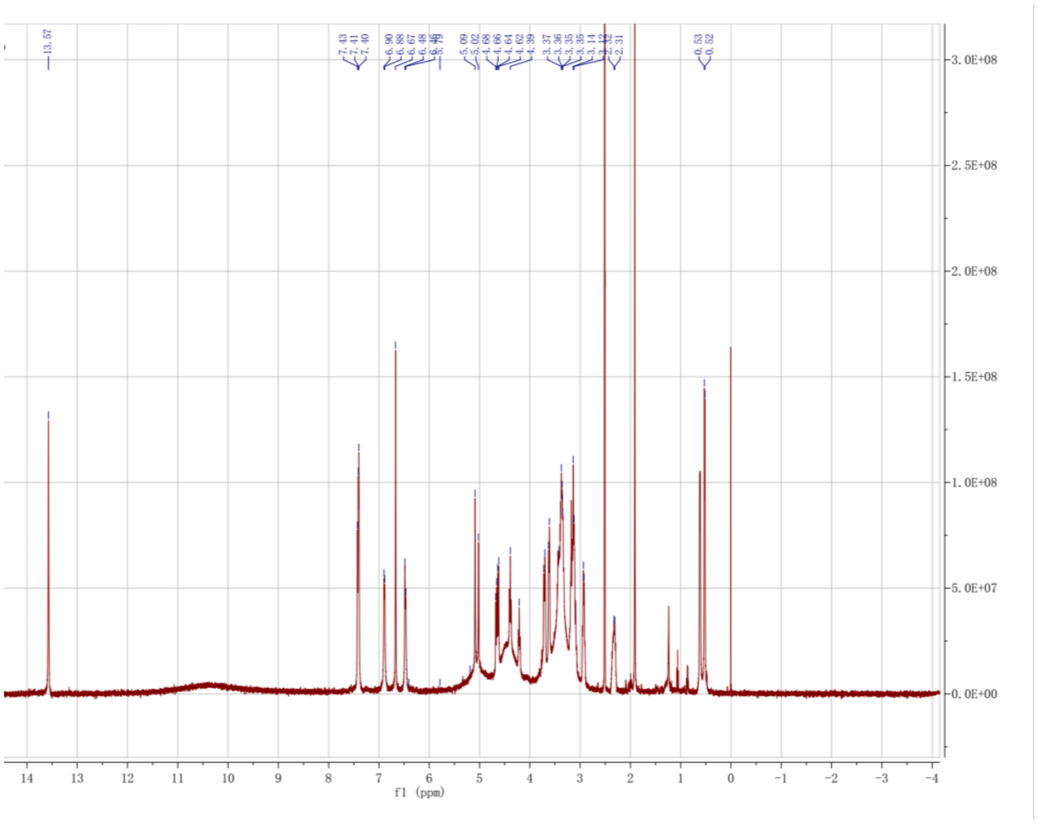

^{13}C-NMR

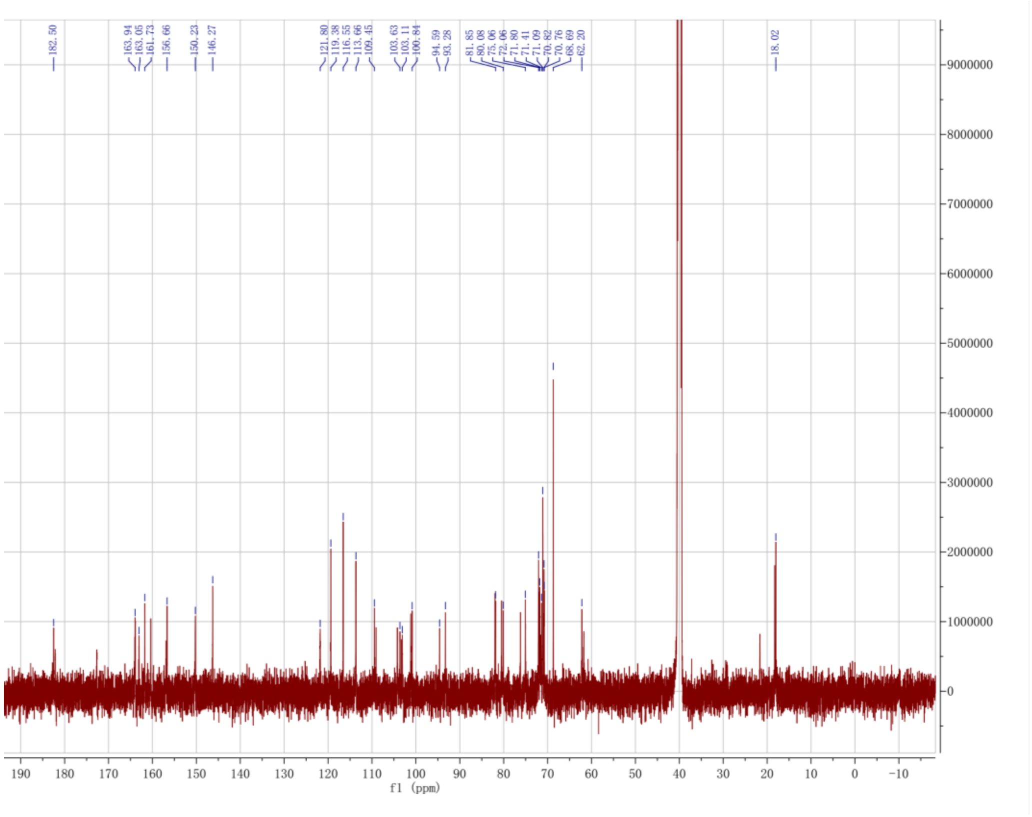

*Compound **b*** Luteolin 6-*C*-glucoside (isoorientin)

1H-NMR

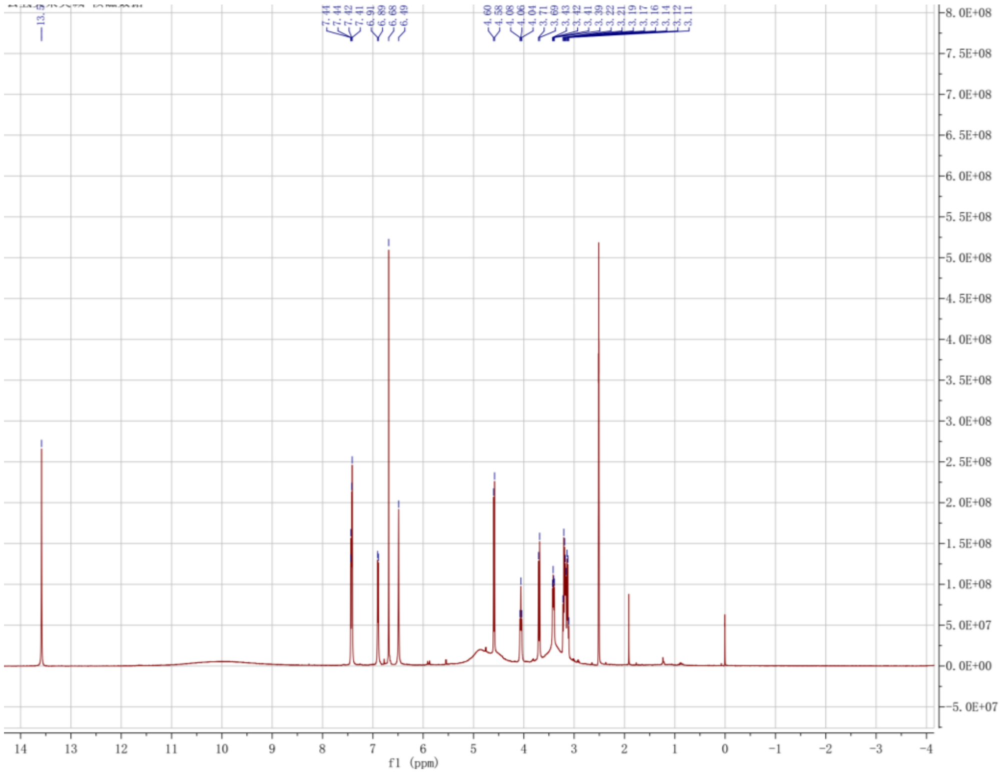

13C-NMR

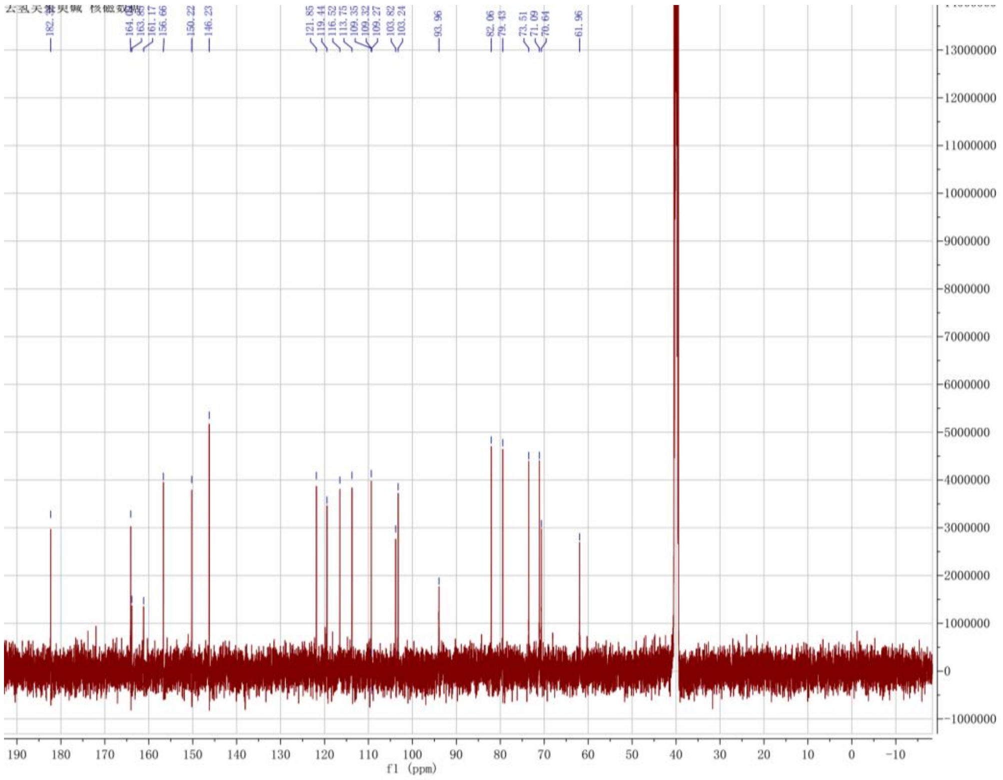

*Compound **c*** Luteolin 8-*C*-glucoside (orientin)

^{1}H-NMR

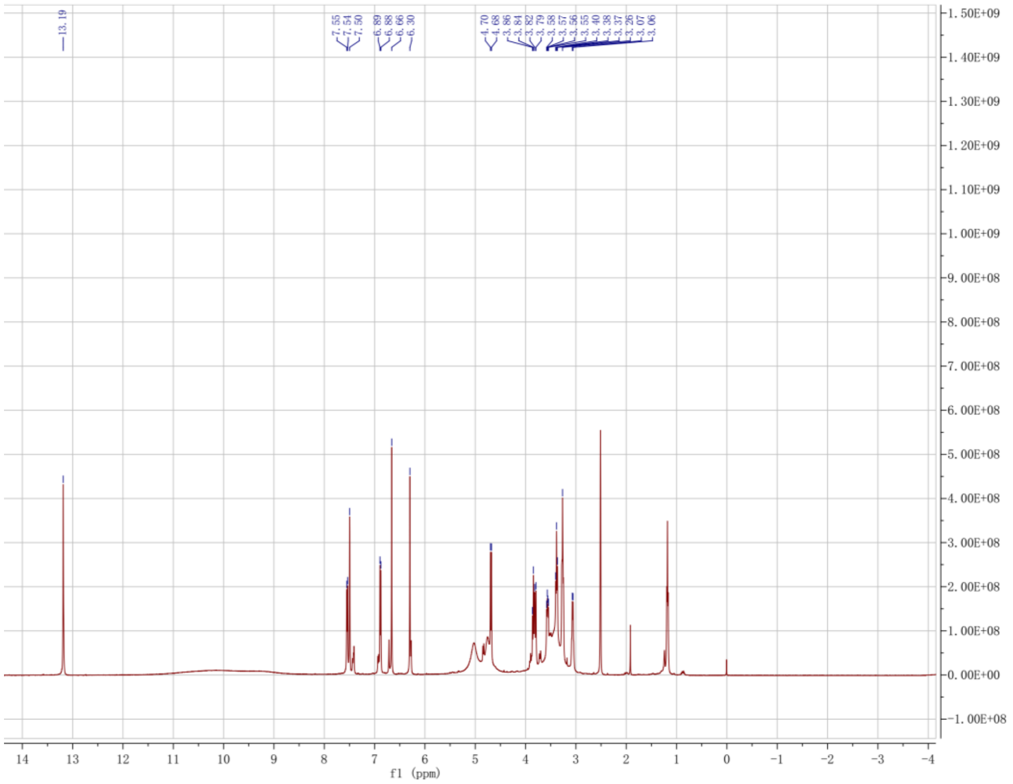

^{13}C-NMR

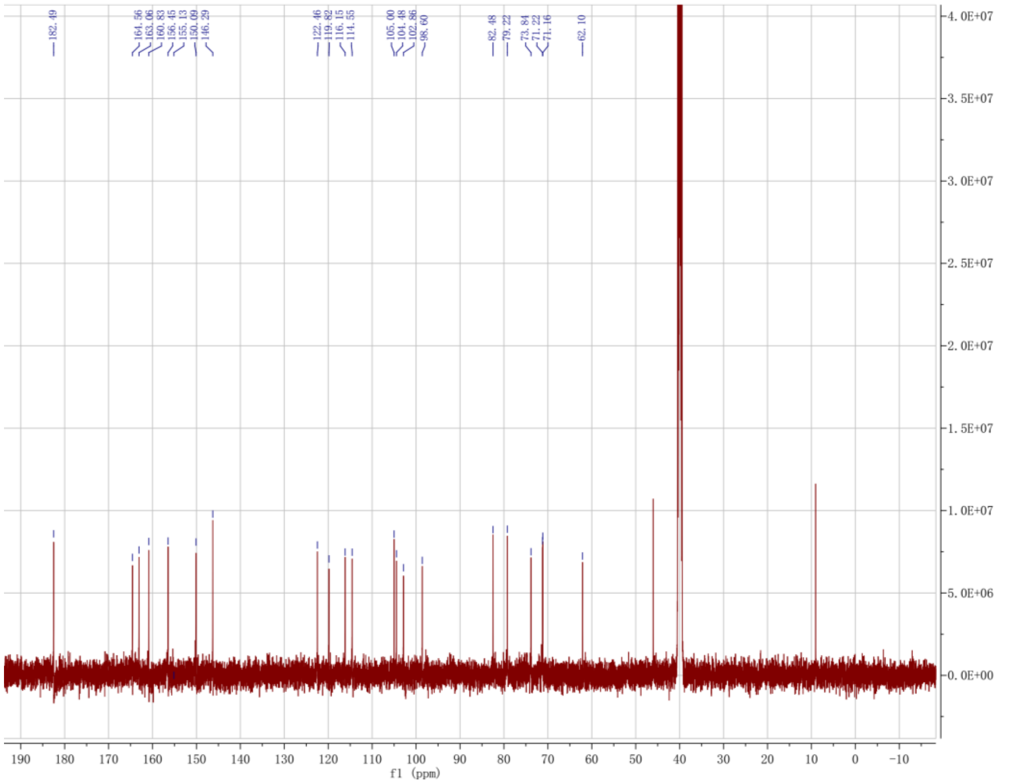

Compound ***d*** Apigenin 8-*C*-neohesperidoside (vitexin-2″-*O*-rhamnoside)

^{1}H-NMR

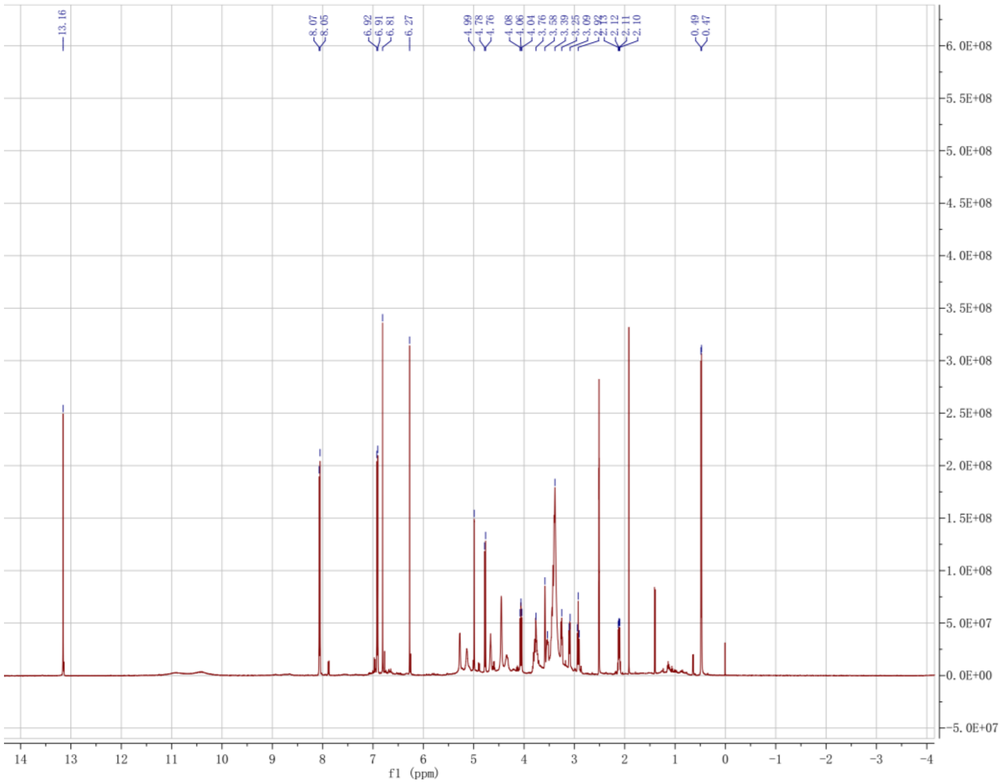

^{13}C-NMR

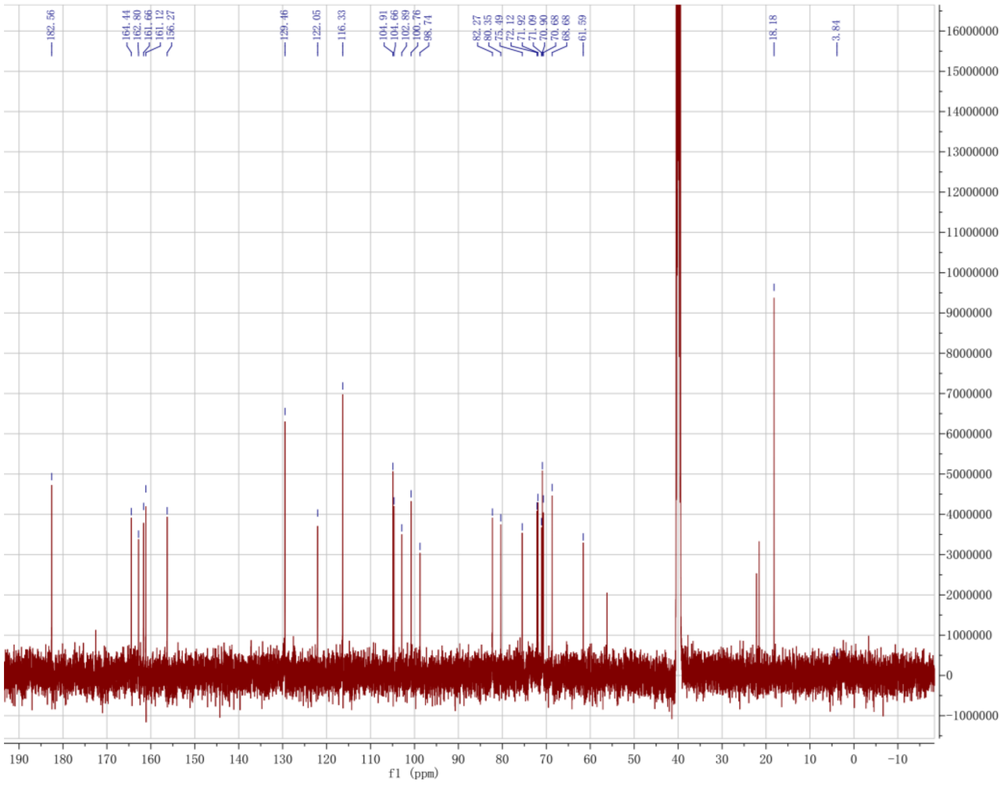

*Compound **e***   Apigenin 6-*C*-neohesperidoside (isovitexin-2″-*O*-rhamnoside)

^{1}H-NMR

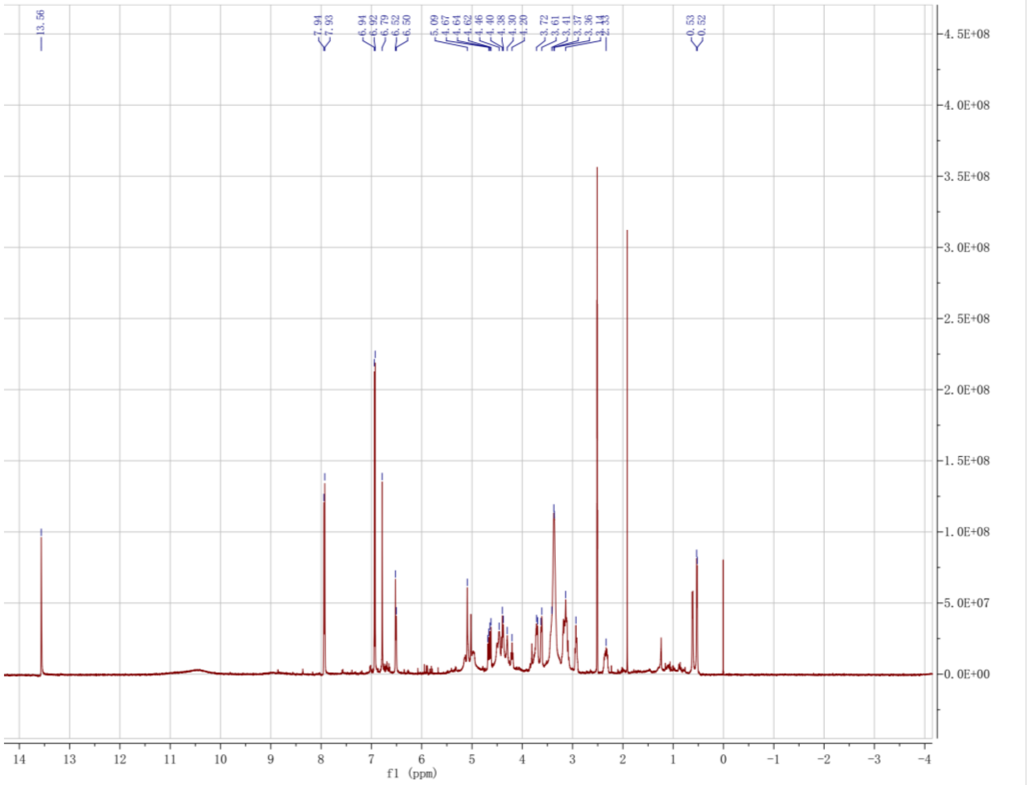

^{13}C-NMR

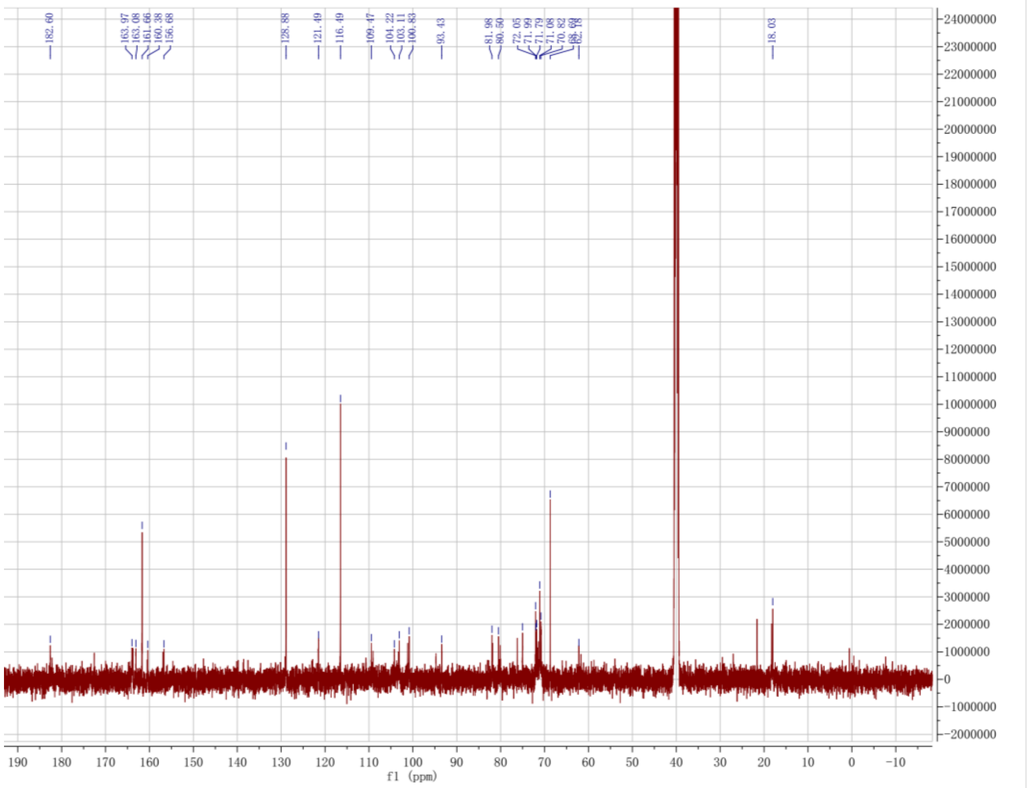

Compound f Apigenin 6-*C*-glucoside (isovitexin)

^{1}H-NMR

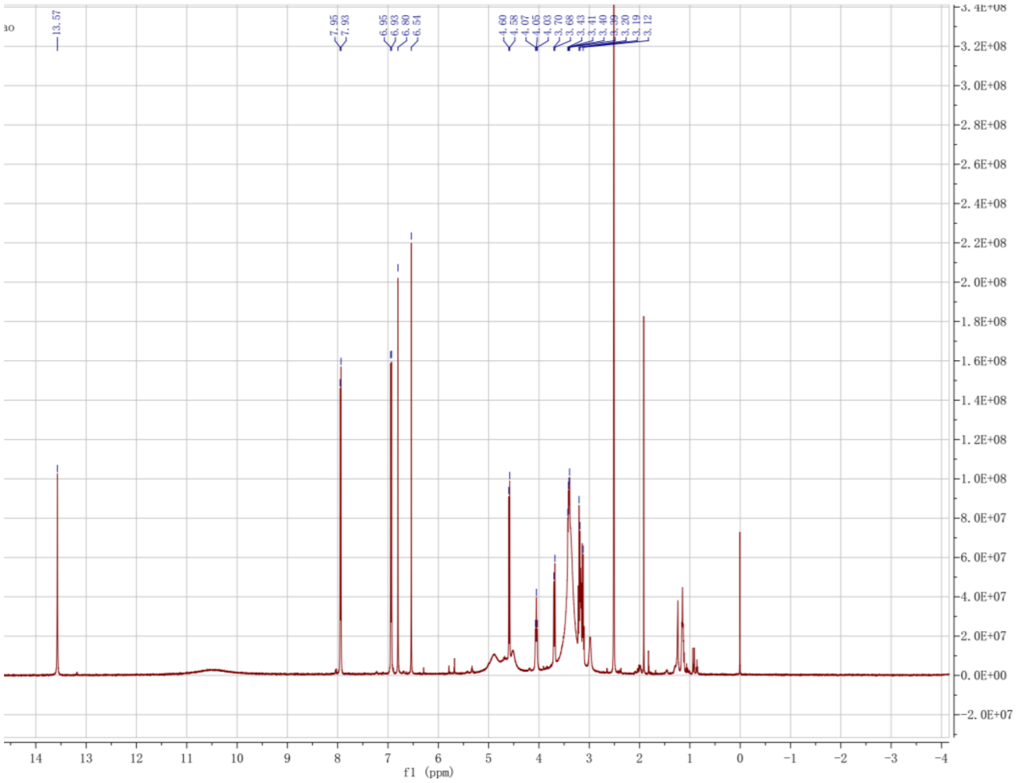

^{13}C-NMR

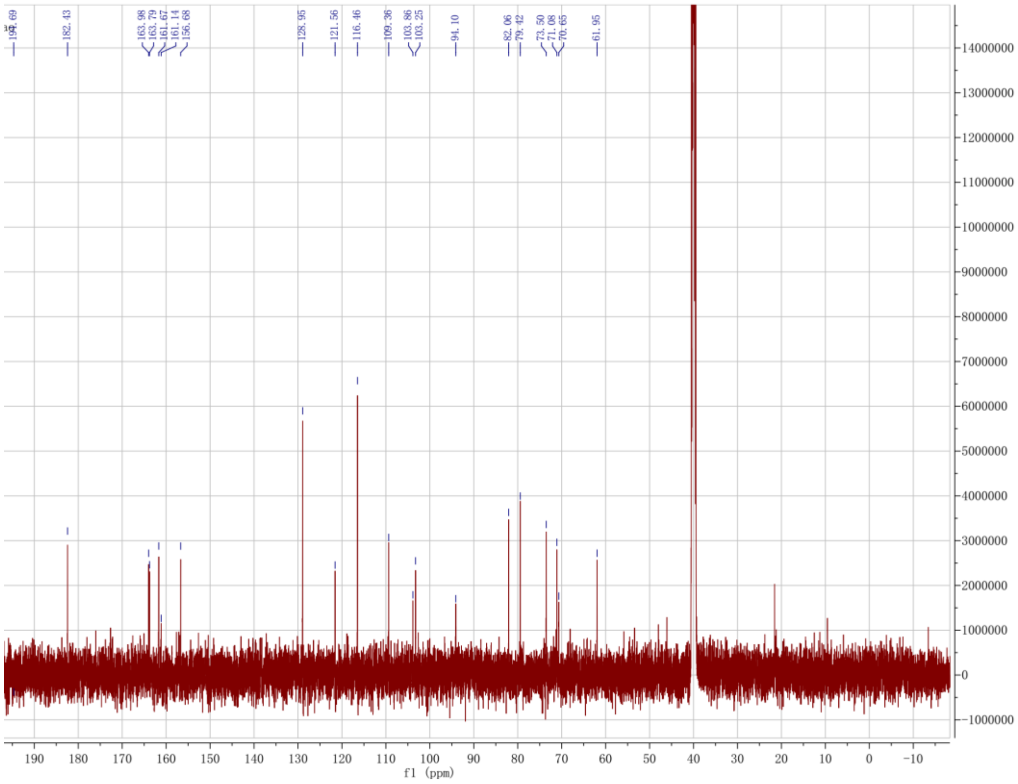

Compound g Diosmetin 7-*O*-rutinoside (diosmin)

^{1}H-NMR

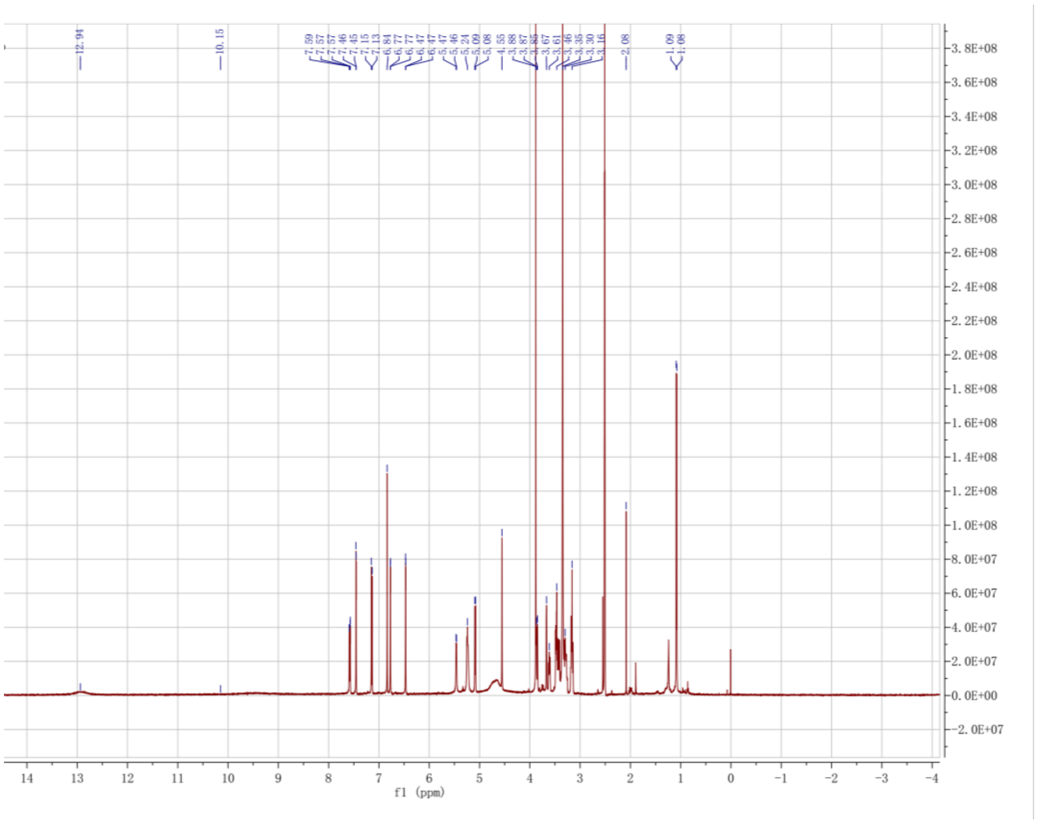

^{13}C-NMR

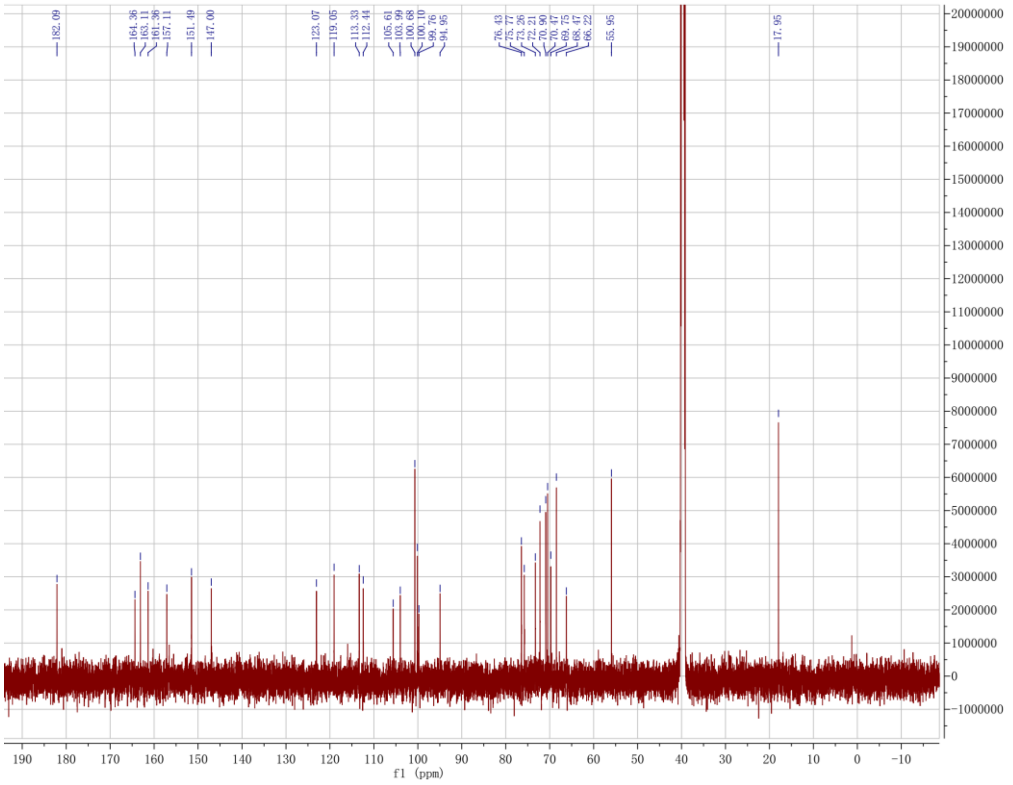

Compound ***h*** Acacetin 7-*O*-rutinoside (linarin)

^{1}H-NMR

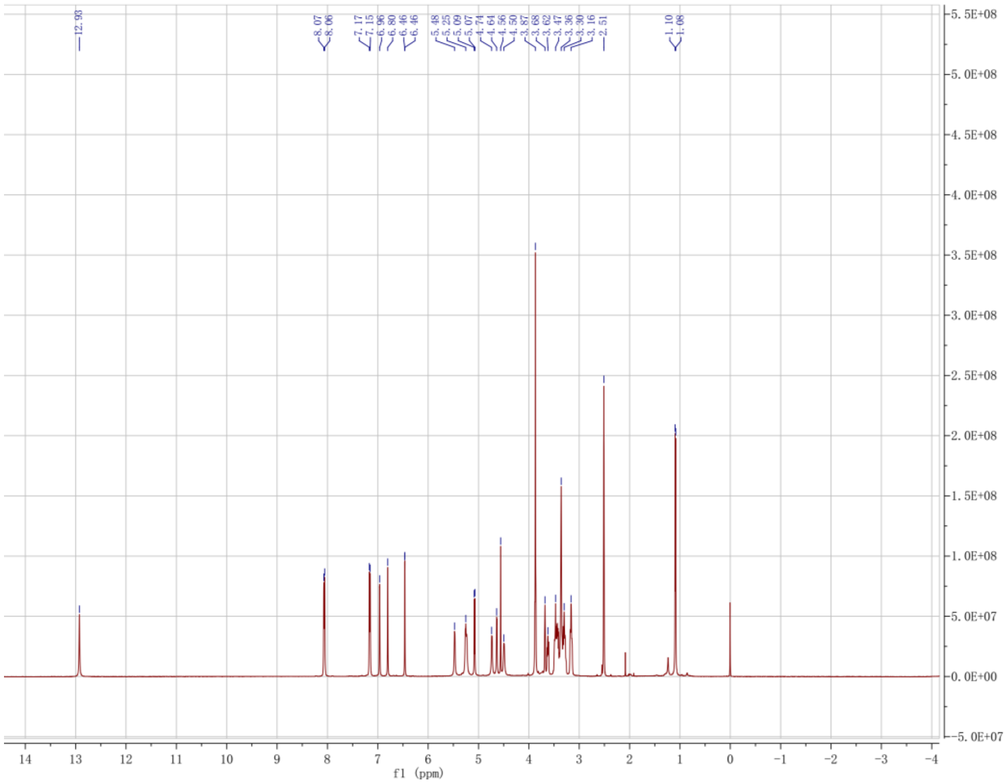

^{13}C-NMR

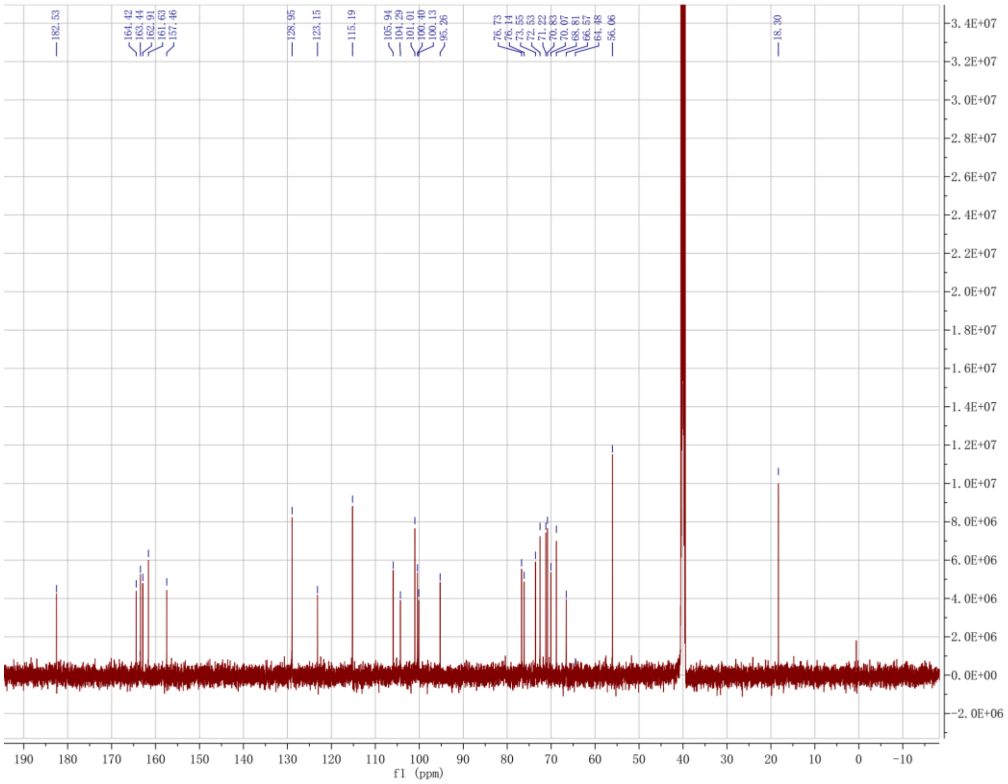

Table S1. Assignments of the ^{1}H- and ^{13}C-NMR (500/100 MHz, DMSO-d6) spectra of *compounds* ***a*-*h***.

No.	*Compound **a***		*Compound **b***		*Compound **c***		*Compound **d***		*Compound **e***		*Compound **f***		*Compound **g***		*Compound **h***	
	δH(J in Hz)	*δC*	*δH(J in Hz)*	*δC*	*δH(J in Hz)*	*δC*	*δH(J in Hz)*	*δC*	*δH(J in Hz)*	*δC*	*δH(J in Hz)*	*δC*	*δH(J in Hz)*	*δC*	*δH(J in Hz)*	*δC*
	**Luteolin**		**Luteolin**		**Luteolin**		**Apigenin**		**Apigenin**		**Apigenin**		**Diosmetin**		**Acacetin**	
2	-	163.0	-	163.9	-	163.1	-	164.4	-	163.9	-	164.0		164.4	-	164.4
3	6.67, s	103.1	6.68, s	103.2	6.66, s	102.9	6.81, s	102.9	6.79, s	103.1	6.80, s	103.3	6.84, s	104.0	6.96, s	104.3
4	-	182.5	-	182.3	-	182.5	-	182.6	-	182.6		182.4		182.1, 55.9(OCH$_3$)	-	182.5
5	13.57, s(OH)	161.7	13.58, s(OH)	161.2	13.19, s(OH)	160.8	13.16, s (OH)	161.1	13.56, s (OH)	161.0	13.57,s (OH)	161.1	12.92, s(OH)	161.4	12.93, s(OH)	162.9
6	-	109.5	-	109.4	6.30, s	98.6	6.27, s	98.7	-	109.5		109.4	6.47, d (2.0)	99.8	6.46, d (1.5)	101.0
7	-	163.9	-	164.1	-	164.6	-	162.8	-	163.1	-	163.8	-	163.1	-	163.4
8	6.47, d (8.5)	94.6	6.49, s	94.0	-	104.5	-	104.7	6.51, d (9.0)	93.4	6.54, s	94.1	6.77, d (2.0)	94.9	6.80, s	95.3
9	-	156.7	-	156.7	-	156.5	-	156.3	-	156.7	-	156.7	-	157.1	-	157.5
10	-	103.6	-	103.8	-	105.0	-	104.9	-	104.2	-	103.9	-	105.6	-	105.9
1'	-	121.8	-	121.8	-	122.5	-	122.0	-	121.5	-	121.6	-	123.1	-	123.2
2'	7.40, d (5.0)	113.7	7.41, d (5.0)	113.6	7.50, s	114.6	8.05, d (8.5)	129.5	7.93, d (8.5)	128.9	7.94, d (9.0)	128.9	7.45, d(2.0)	113.3	8.06, d (7.5)	128.9
3'	-	146.3	-	146.2	-	146.3	6.91, d (9.0)	116.3	6.93, d (9.0)	116.5	6.93, d (8.5)	116.5	-	147.0	7.15, d (8.0)	115.2
4'	-	150.2	-	150.2	-	150.1	-	161.7	-	161.7	-	161.7	3.88(OCH$_3$)	151.5	3.87(OCH$_3$)	161.6, 56.1(OCH$_3$)
5'	6.89, d(8.0)	116.5	6.89, d (8.5)	116.5	6.88, d (8.5)	116.2	6.91, d (9.0)	116.3	6.93, d (9.0)	116.5	6.93, d (8.5)	116.5	7.14, d (8.5)	112.4	7.15, d (8.0)	115.2
6'	7.42, s	119.4	7.44, d(2.0)	119.4	7.54, d (9.0)	119.8	8.05, d (8.5)	129.5	7.93, d (8.5)	128.9	7.94, d (9.0)	128.9	7.58, dd(2.0)	119.1	8.06, d (7.5)	128.9
	**Glu**		**Glu**		**Glu**		**Glu**		**Glu**		**Glu**		**Glu**		**Glu**	
1''	4.62	72.0	4.59	73.5	4.68	73.8	4.76	72.1	4.65	72.0	4.58,	73.5	4.59	100.1	4.55	100.4
2''	4.21	75.1	4.06	70.6	3.83	71.2	4.06	75.5	4.20	75.0	4.05	71.1	3.85	73.3	3.87	73.6
3''	3.37	80.1	3.21	79.4	3.27	79.23	.39	80.4	3.37	80.5	3.19	79.4	3.20	76.4	3.62	76.1
4''	3.62	70.8	3.41	71.1	3.38	71.1	3.35	70.9	3.62	70.8	3.39	70.6	3.35	72.2	3.35	72.5
5''	3.14	81.8	3.13	82.1	3.06	82.5	3.08	82.3	3.18	82.0	3.13	82.1	3.42	75.8	3.47	76.7
6''	3.70, 3.42	62.1	3.71, 3.68	62.0	3.79, 3.57	62.1	3.76, 3.58	61.6	3.72, 3.41	62.2	3.69, 3.41	62.0	3.69, 3.35	66.2	3.68, 3.35	66.6
	**Rha**						**Rha**		**Rha**				**Rha**		**Rha**	
1'''	5.05	100.8					4.99	100.8	5.09	100.8			5.45	100.7	5.48	100.1
2'''	3.12	71.5					3.42	71.1	3.14	71.8			3.16	70.9	3.16	71.2
3'''	3.36	71.1					3.25	70.7	3.36	71.1			3.30	70.5	3.30	70.1
4'''	2.92	71.8					2.92	71.9	2.93	72.1			3.35	69.7	3.35	70.8

5‴	2.32	68.7	2.11	68.7	2.33	68.7	2.09	68.5	2.08	68.8
6‴	0.52	18.0	0.48	18.2	0.52	18.0	1.08	17.9	1.08	18.3

Mass spectrometry analysis

2.2.1. Characterization of flavone C-glycosides

Peaks **2**, **3**, **4**, **6**, **7**, and **9** were definitively identified as luteolin 6-*C*-neohesperidoside, luteolin 6-*C*-glucoside, luteolin 8-*C*-glucoside, apigenin 8-*C*-neohesperidoside, apigenin 6-*C*-neohesperidoside, and apigenin 6-*C*-glucoside by comparing their precise molecular weights, retention times, ultraviolet spectra, and fragment ions with those of the reference compounds (obtained via HSCCC-prep-HPLC).

Peak **5** showed a deprotonated ion $[M–H]^-$ at *m*/*z* 593 ($C_{27}H_{30}O_{15}$) and MS^2 fragment ions at *m*/*z* 447 (loss of Rha, 146 Da), *m*/*z* 357 $[^{0,3}X]^-$, *m*/*z* 327 $[^{0,2}X]^-$, *m*/*z* 285, *m*/*z* 163, and *m*/*z* 133, which are consistent with luteolin 6-*C*-neohesperidoside (*Peak* **2**). The only difference was the ion ratio at 357/327, which is less than 1/5, indicating that *C*-8 was conjugated. Therefore, *Peak* **5** was assigned to luteolin 8-*C*-neohesperidoside.

Peak **8** showed the same deprotonated ion $[M–H]^-$ (*m*/*z* 593, $C_{27}H_{30}O_{15}$) as *Peak* **5**, but there were several discrepancies. Firstly, the fragment ion at *m*/*z* 431 indicates the loss of a glucoside (Glc, 162 Da). The MS^2 ions at *m*/*z* 341 $[^{0,3}X]^-$ and *m*/*z* 311 $[^{0,2}X]^-$ are typical of *C*-flavones, and the ratio 341/311 is less than 1/5, indicating the presence of *C*-8 conjugation. The generated ions at *m*/*z* 283, *m*/*z* 269, *m*/*z* 175, *m*/*z* 131, and *m*/*z* 117 suggest that apigenin was the parent flavone. *Peak* **8** was speculated to be apigenin 8-*C*-diglucoside.

Peak **15** showed a deprotonated ion $[M–H]^-$ at *m*/*z* 433 ($C_{21}H_{22}O_{10}$), and the MS^2 ions at *m*/*z* 343 $[^{0,3}X]^-$, *m*/*z* 313 $[^{0,2}X]^-$, and *m*/*z* 271 led to the tentative identification of naringenin *C*-glucoside.

2.2.2. Characterization of Flavones and Flavone O-glycosides

The discrimination of flavone types is an essential basis for the identification of flavone *O*-glycosides and other derivatives in OHPE. The data reported in the literature are also critical to the process of identification because they serve as references for comparison. Flavones were usually cleaved at the C ring, and fracture modes were differentiated by the substituent types and positions on the A and B rings. The main splitting mechanism of flavones in OHPL was $[^{1,3}A]^-$ accompanied by $[^{1,3}B]^-$ and $[^{0,4}B]^-$ fractures as shown in Figure 3.

Aromadendrin [1] (*Peak* **19**), kaempferol [1] (*Peak* **26**), naringenin [1] (*Peak* **35**), and apigenin [1] (*Peak* **38**) were confirmed to be present in OHP, and their identities were assigned after comprehensive analysis by the analytical comparison of accurate molecular weights, UV spectra, retention behaviors, splitting types, and relative abundance of the fragment ions with the data in the literature [1]. Luteolin (*Peak* **32**), acacetin (*Peak* **37**), and diosmetin (*Peak* **43**) were also used as the basis for identifying flavone derivatives in OHPE in the present study. Flavone isomers were found in abundance.

Peak **18** ($C_{15}H_{12}O_5$) had a deprotonated ion at *m*/*z* 271, which is consistent with naringenin. It was tentatively characterized as a naringenin isomer according to the accurate molecular weight.

Peak **27** ($C_{15}H_{12}O_6$) showed a deprotonated ion at *m*/*z* 287 $[M–H]^-$ and MS^2 ions at *m*/*z* 151 $[^{1,3}A]^-$ and *m*/*z* 135 $[^{1,3}B]^-$, which are in accordance with aromadendrin, but the relative abundance of the *m*/*z* 135 $[^{1,3}B]^-$ fracture was slightly higher than that of aromadendrin. *Peak* **27** was speculated to be eriodictyol.

Peak **31** ($C_{16}H_{12}O_5$) exhibited a deprotonated ion at *m*/*z* 287 $[M–H]^-$ and MS^2 ions at *m*/*z* 151 $[^{1,3}A]^-$ and *m*/*z* 131 $[^{1,3}B]^-$, which are consistent with acacetin. Thus, it was tentatively characterized as an acacetin isomer according to the accurate molecular weight.

Peak **44** ($C_{16}H_{12}O_6$) had a deprotonated ion at *m*/*z* 299, which is in accordance with diosmetin. It was preliminarily identified as a diosmetin isomer.

Flavone *O*-glycosides easily lost the glycoside or diglycoside moiety in MS^2, even at the fragmentor voltage (135 V) of MS mode.

Peaks **17** and **19** were proved definitively to be diosmetin 7-*O*-rutinoside and acacetin 7-*O*-rutinoside, respectively. The rutinoside moiety was very easily removed to generate the parent ion.

Peak **1** ($C_{27}H_{32}O_{15}$) showed a deprotonated ion at *m/z* 595 $[M–H]^-$ and UV spectra at 287 nm. The main MS^2 ion at *m/z* 287 indicates the loss of rutinoside, and the MS^2 ions at *m/z* 151 $[^{1,3}A]^-$ and *m/z* 135 $[^{1,3}B]^-$ are in accordance with aromadendrin. Therefore, *Peak* **1** was tentatively identified as aromadendrin 3-*O*-rutinoside.

Peaks **10**, **28**, and **30**, similar to flavone *C*-glycosides (apigenin 8-*C*-neohesperidoside and apigenin 6-*C*-neohesperidoside), appeared to have the same chemical formula ($C_{27}H_{30}O_{14}$) on the basis of the deprotonated ion at exactly *m/z* 577 $[M–H]^-$, implying that these compounds were isomers. However, these three peaks easily lost diglycosides, and there were no characteristic fragments of flavone *C*-glycosides. This suggests that they were flavone *O*-glycosides. They also produced some different MS ions.

Peak **10** showed MS^2 ions at *m/z* 431 $[M–H–Rha]^-$ and *m/z* 269 $[M–H–Rha–Glc]^-$, with the latter predominating, suggesting the conjugation of rutinoside. The ions at *m/z* 151 $[^{1,3}A]^-$ and *m/z* 117 $[^{1,3}B]^-$ suggest that apigenin was the aglycone. Therefore, it was speculated to be apigenin 7-*O*-rutinoside. Similarly, *Peak* **16** was identified as apigenin 7-*O*-glucoside.

Peaks **28** and **30** showed consistent MS^2 ions at *m/z* 445 and 283, indicating the loss of a pentoside (132 Da) and Glc (162 Da), respectively. The typical ions at *m/z* 151 $[^{1,3}A]^-$ and *m/z* 131 $[^{1,3}B]^-$ suggest that acacetin was the aglycone. Both peaks were tentatively identified as acacetin *O*-glc-pentoside.

Peak 12 ($C_{28}H_{36}O_{13}$) had a deprotonated ion at *m/z* 579 $[M–H]^-$, and the MS^2 ions at *m/z* 417 and *m/z* 271 suggest the loss of Glc (162 Da) and Rha (146 Da), respectively. The typical ions at *m/z* 151 $[^{1,3}A]^-$ and *m/z* 119 $[^{1,3}B]^-$ suggest that naringenin was the base. *Peak* 12 was tentatively identified as naringenin *O*-rha-glucoside.

2.2.3. Characterization of Isoflavones and Isoflavone O-glycosides

Isoflavones are another important flavone isomer in OHPE. Similar to flavones, the main fragments of isoflavones were also generated by splitting of the C ring, and the MS^2 ions $[^{1,3}A]^-$ and $[^{1,3}B]^-$ were similar to those of flavones. However, the MS^2 ions $[M–H–CO]^-$ and $[M–H–2CO]^-$ have been shown to occur in abundance for isoflavones [2, 3]. From this key difference between isoflavones and flavones, combined with previously reported data, biochanin A [1] (*Peak* **20**), daidzein [1] (*Peak* **25**), genistein [1] (*Peak* **36**), isoformononetin [1] (*Peak* **45**), and isoprunetin [1] (*Peak* **46**) were assigned, as shown in Table 2.

Peak **11** ($C_{21}H_{20}O_{10}$) had a deprotonated ion at *m/z* 431 $[M–H]^-$ and MS^2 ion at *m/z* 269, indicating the loss of Glc (162 Da), and the typical MS^2 ions at *m/z* 241 $[M–H–CO]^-$, *m/z* 213 $[M–H–2CO]^-$, *m/z* 151 $[^{1,3}A]^-$, and *m/z* 117 $[^{1,3}B]^-$ indicate a genistein-based aglycone. It was tentatively identified as genistein7-*O*-glucoside. Similarly, *Peak* **13** was identified as genistein7-*O*-rutinoside.

Peak **14** showed a protonated ion at *m/z* 593 $[M+H]^+$ ($C_{28}H_{32}O_{14}$) and MS^2 fragment ion at *m/z* 285 (loss of rutinoside, 308 Da). The other fragment ions are consistent with biochanin A, so *Peak* **14** was tentatively identified as biochanin A-*O*-rutinoside.

Peak **21** ($C_{27}H_{30}O_{14}$) contained a deprotonated ion at *m/z* 577 $[M–H]^-$. The MS^2 fragment ions at *m/z* 445 and *m/z* 283 indicate the loss of a pentoside (132 Da) and Glc (162 Da), respectively. MS^2 fragment ions at *m/z* 255 $[M–H–CO]^-$, *m/z* 227 $[M–H–2CO]^-$, *m/z* 165 $[^{1,3}A]^-$, and *m/z* 117 $[^{1,3}B]^-$ suggest that isoprunetin was the parent flavone. Therefore, *Peak* **21** was speculated to be isoprunetin 7-*O*-glc-pentoside. Similarly, *Peak* **33** was assigned as isoprunetin 7-*O*-glucoside.

2.2.4. Characterization of Prenylflavones and Polymethoxyflavones (PMFs)

Prenylflavones were confirmed to be present in OHP in a previous report [1]. Because of the low abundance in our present study, two prenylflavones were speculated to be present according to their precise molecular weights. *Peak* **23** ($C_{20}H_{20}O_5$) showed a deprotonated ion at precisely *m/z* 339.1247 (−2.2 ppm). From values in the SciFinder database, *Peak* **23** was tentatively identified as prenylnaringenin. Similarly, *Peak* **34** ($C_{20}H_{20}O_6$), with a deprotonated ion at exactly *m/z* 355.1196 (0.37 ppm), was tentatively identified as prenylaromadendrin.

PMFs, with polymethoxy substituents on the parent nucleus, are known to easily lose radical *n*×CH_3 and neutral ions (such as CO and H_2O) [4]. In this investigation, traces of PMFs were detected, and all of them were tentatively identified according to the accurate molecular weight and predominant MS^2 ions. *Peaks* ***20*** and ***24*** were estimated to be pentamethoxyflavanone and dimethoxyl-dihydroxyflavone in positive ion mode. *Peaks* ***39*** and ***40*** showed the same deprotonated ion at *m*/*z* 297 ($C_{17}H_{14}O_5$), suggesting that they are isomers, and dimethoxyl-hydroxyflavone was assigned. *Peaks* ***41*** and ***42*** both had a deprotonated ion at *m*/*z* 273 ($C_{19}H_{18}O_8$), and both of them were preliminarily identified as tetramethoxyl-dihydroxyflavone.

References

1. Feng, S.X.; Hao, J.; Xu, Z.F.; Chen,T.; Qiu, S.X. Polyprenylated isoflavanone and isoflavonoids from Ormosia henryi and their cytotoxicity and anti-oxidation activity. *Fitoterapia* **2012,** 83, (1), 161-165.
2. Kang, J.; Hick, L. A.; Price, W. E. A fragmentation study of isoflavones in negative electrospray ionization by MSn ion trap mass spectrometry and triple quadrupole mass spectrometry. *Rapid Commun. Mass Spectr.* **2007,** 21, (6), 857-868.
3. Nakata, R.; Yoshinaga, N.; Teraishi, M.; Okumoto, Y.; Huffaker, A.; Schmelz A.; Mori, N. A fragmentation study of isoflavones by IT-TOF-MS using biosynthesized isotopes. *Biosci. Biotech. Bioch.* **2018**.
4. Xing, T.T.; Zhao, X.J.; Zhang, Y.D.; Li, Y.F. Fast Separation and Sensitive Quantitation of Polymethoxylated Flavonoids in the Peels of Citrus Using UPLC-Q-TOF-MS. *J. Agric. Food Chem.* **2017,** 65, (12), 2615-2627.
